# Perturbed hematopoietic stem and progenitor cell hierarchy in myelodysplastic syndromes patients with monosomy 7 as the sole cytogenetic abnormality

**DOI:** 10.18632/oncotarget.12234

**Published:** 2016-09-24

**Authors:** Marios Dimitriou, Petter S. Woll, Teresa Mortera-Blanco, Mohsen Karimi, David C. Wedge, Helen Doolittle, Iyadh Douagi, Elli Papaemmanuil, Sten Eirik W. Jacobsen, Eva Hellström-Lindberg

**Affiliations:** ^1^ Center for Hematology and Regenerative Medicine, Karolinska Institutet, Department of Medicine, Karolinska University Hospital Huddinge, Stockholm, Sweden; ^2^ Haematopoietic Stem Cell Biology Laboratory, MRC Molecular Haematology Unit, Weatherall Institute of Molecular Medicine, University of Oxford, Oxford, United Kingdom; ^3^ Cancer Genome Project, Wellcome Trust Sanger Institute, Hinxton, United Kingdom; ^4^ Oxford Big Data Institute, Li Ka Shing Centre for Health Information and Discovery, Wellcome Trust Centre for Human Genetics Oxford, United Kingdom; ^5^ Computational Oncology, Epidemiology and Biostatistics Memorial Sloan Kettering Cancer Institute, New York, NY, United States of America

**Keywords:** cancer stem cells, monosomy 7, myeloid leukemia, myelodysplastic syndrome, azacitidine

## Abstract

The stem and progenitor cell compartments in low- and intermediate-risk myelodysplastic syndromes (MDS) have recently been described, and shown to be highly conserved when compared to those in acute myeloid leukemia (AML). Much less is known about the characteristics of the hematopoietic hierarchy of subgroups of MDS with a high risk of transforming to AML. Immunophenotypic analysis of immature stem and progenitor cell compartments from patients with an isolated loss of the entire chromosome 7 (isolated −7), an independent high-risk genetic event in MDS, showed expansion and dominance of the malignant −7 clone in the granulocyte and macrophage progenitors (GMP), and other CD45RA^+^ progenitor compartments, and a significant reduction of the LIN^−^CD34^+^CD38^low/−^CD90^+^CD45RA^−^ hematopoietic stem cell (HSC) compartment, highly reminiscent of what is typically seen in AML, and distinct from low-risk MDS. Established functional *in vitro* and *in vivo* stem cell assays showed a poor readout for −7 MDS patients irrespective of marrow blast counts. Moreover, while the −7 clone dominated at all stages of GM differentiation, the −7 clone had a competitive disadvantage in erythroid differentiation. In azacitidine-treated −7 MDS patients with a clinical response, the decreased clonal involvement in mononuclear bone marrow cells was not accompanied by a parallel reduced clonal involvement in the dominant CD45RA^+^ progenitor populations, suggesting a selective azacitidine-resistance of these distinct −7 progenitor compartments. Our data demonstrate, in a subgroup of high risk MDS with monosomy 7, that the perturbed stem and progenitor cell compartments resemble more that of AML than low-risk MDS.

## INTRODUCTION

Myelodysplastic syndromes (MDS) constitute a heterogeneous group of hematological clonal disorders, characterized by ineffective hematopoiesis and cytopenias, as well as a high but varying risk for transformation to acute myeloid leukemia (AML) [1]. Cytogenetic abnormalities contribute not only to the risk estimation in MDS [2, 3] but may also reflect specific biological features and targets for therapy, the best example being the response to lenalidomide in patients with an isolated deletion of chromosome 5q arm (del(5q)) and less than 5% marrow blasts [4, 5]. By contrast, abnormalities encompassing chromosome 7, del(7q) and monosomy 7 (−7), are associated with intermediate and high risk for transformation, respectively, both before [2, 6-8], and after allogeneic stem cell transplantation [9, 10]. Although complete or partial loss of chromosome 7 is frequently found together with other cytogenetic lesions as well as with recurrent mutations [11], it appears to represent an independent high-risk factor, and hence a potential target for therapy [7, 8].

A major current focus in cancer research is to identify and characterize the cancer stem cells (CSCs) capable of propagating the disease [12-14]. Although early studies in AML patients suggested that only CD34^+^CD38^−^ cells retained AML-propagating properties [15, 16], more recent studies have revealed that cells outside this compartment may also have cancer propagating potential [17-20]. Whereas the stem and progenitor cell compartments are severely perturbed in AML [18], several studies focusing on low to intermediate-risk MDS patients have established remarkably well preserved compartments of hematopoietic stem cells (HSCs) and myelo-erythroid progenitor compartments, as assessed phenotypically [21-25] and transcriptionally [25, 26], despite being highly clonally involved at quite unperturbed frequencies [25, 27]. Through such studies, LIN^−^CD34^+^CD38^−^CD90^+^CD45RA^−^ HSCs have been strongly implicated as the cellular origin of MDS stem cells [25, 27-30]. Moreover, back-tracking of diverse genetic lesions to the distinct LIN^−^CD34^+^CD38^−^CD90^+^CD45RA^−^ MDS stem cells strongly implicate that the cancer propagating potential in del(5q) low-intermediate risk MDS, exclusively resides within rare and distinct LIN^−^CD34^+^CD38^−^CD90^+^CD45RA^−^ MDS stem cells, and that these outcompete the normal HSC compartment [25].

The HSCs and myelo-erythroid progenitor compartments have only been investigated in a low number of higher-risk MDS patients [24], and thus it remains to be determined to what degree the hematopoietic stem-progenitor cell hierarchy in higher-risk MDS is distinct from that in low- intermediate-risk MDS [25], and whether it more resembles that typically observed in AML [18]. Herein, this was addressed in intermediate to high -risk MDS patients with isolated monosomy 7.

## RESULTS

### Suppression of the LIN^−^CD34^+^CD38^low/−^CD90^+^ stem cell compartment and expansion of CD45RA^+^ progenitors in isolated −7 MDS patients

**Figure 1:**
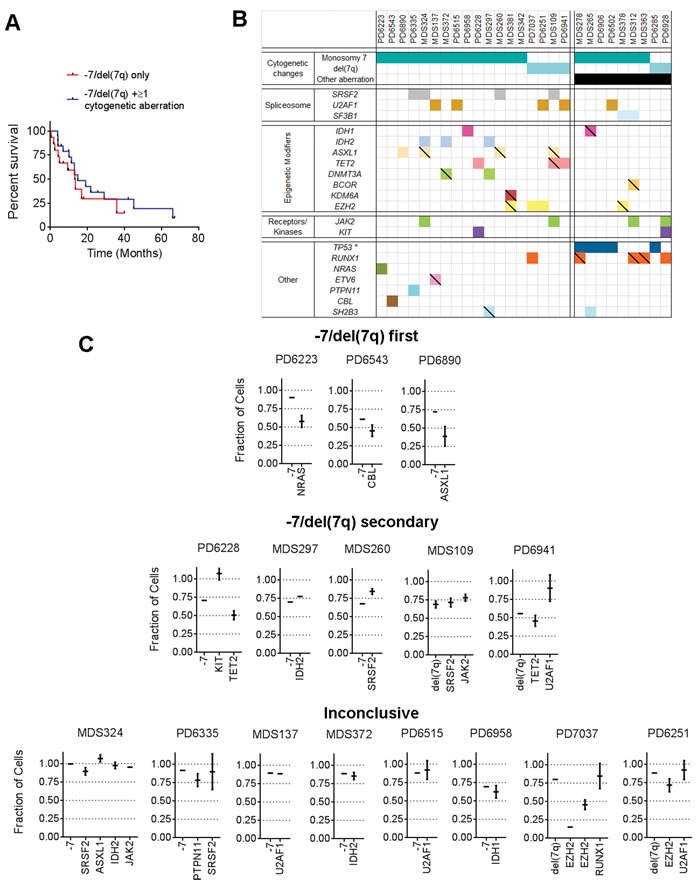
Co-occurrence of chromosome 7 abnormalities and recurrent driver mutations **A.** Survival (Kaplan Meier) after diagnosis of MDS patient cohort with chromosome 7 abnormalities grouped as −7/del(7q) only (*n* = 15); or as −7/del(7q) + ≥ 1 cytogenetic aberration (*n* = 20). **B.** Co-occurrence map of −7 and del(7q) with oncogenic mutations (empty boxes) and truncating/unknown mutations (hatched\scored boxes) as described in supplementary methods. **C.** Computational prediction of fraction of cells with specified genetic lesions, within total BM mononuclear cells from patients with isolated −7 or isolated del(7q). Patients were grouped based on predicted hierarchy of genomic lesions. Error bars indicate 95% confidence interval (CI).

Since we in these studies, had limited availability of MDS patients with isolated monosomy 7 from which we were able to collect sufficient bone marrow cells to characterize their hematopoietic stem and progenitor cell hierarchies, we first validated whether our cohort appeared to be clinically and prognostically representative of MDS patients with isolated monosomy 7 as a whole. In agreement with previous studies [7, 8], our MDS cases with either complete or partial loss of chromosome 7 as an isolated cytogenetic aberration (−7/del(7q) only) showed an equally poor survival as patients with −7/del(7q) plus one or more additional cytogenetic aberration (−7/del(7q) + ≥ 1 cytogenetic aberration) [7, 8], with 50% mortality within 15 months from diagnosis (Figure 1A). Targeted sequencing of BM cells from MDS patients with chromosome 7 abnormalities identified a large set of mutations in genes recurrently mutated in MDS [11, 31], (Figure 1B; Supplemental Figure 1). With the limited number of patients included in the analysis, the only significant co-occurrence was between *TP53* mutations and −7/del(7q) aberrations, where all five patients with a *TP53* mutation had at least one more karyotypic abnormality, while none of the 18 patients with isolated −7/del(7q) had detectable *TP53* mutations (Fisher exact **p = 0.004). Moreover, meta-analysis of a published cohort of MDS patients suggested that *TP53* mutations are less common in patients with a complex karyotype without −7/del(7q) (6 out of 34 cases) than in those with a complex karyotype including −7/del(7q) (5 out of 9 cases; (Supplemental Table 2). Computational prediction of isolated −7/del(7q) patients based on targeted sequencing data (Figure 1C; Supplemental Tables 3-4) demonstrated that −7/del(7q) could precede (3 cases) as well as be secondary (5 cases) to other oncogenic mutations, based on a 95% confidence interval. In 8 cases the computational analysis failed to statistically separate the sequential acquisition pattern. Too few patients (*n* = 16) were investigated to be able to establish whether any distinct oncogenic mutations might systematically precede or be secondary to −7/del(7q).

**Figure 2:**
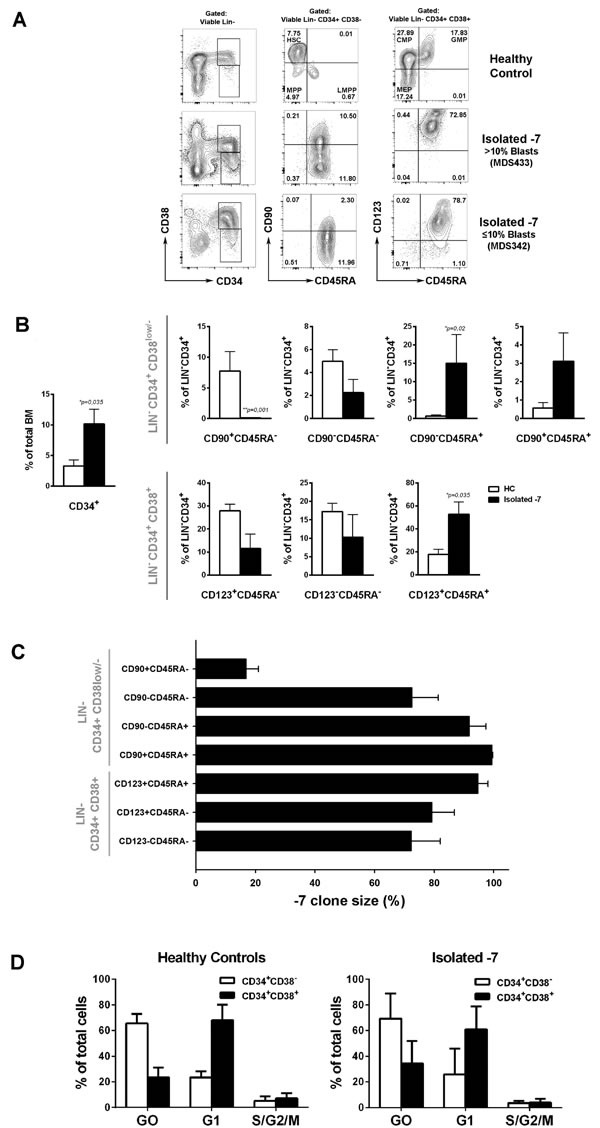
Phenotypic characterization and clonal involvement of stem and progenitor cells in patients with isolated monosomy 7 **A.** FACS profiles of stem and progenitor cell compartments in representative BM mononuclear cells. Numbers indicate percentages of LIN^−^CD34^+^ cells. **B.** Mean (SEM) frequencies within total BM or within total CD34^+^ stem-progenitor cell compartment of healthy controls (HC; *n* = 6) and isolated −7 MDS cases (*n* = 7) *p < 0.05. **C.** Mean (SEM) percentage of cells with −7 as assessed by FISH in FACS-purified stem/progenitor cell populations from isolated −7 patients (*n* = 7). For purified LIN^−^CD34^+^CD38^low/−^CD90^+^CD45RA^−^ cells, 9/ 72 (12,5%) nuclei were scored as monosomy 7 for MDS372 and 12/60 (20%) for MDS381. **D.** Cell cycle analysis from HC (*n* = 4) and isolated −7 (*n* = 4; patients MDS324, MDS260, MDS342 and MDS372) for LIN^−^CD34^+^CD38^−^ or LIN^−^CD34^+^CD38^+^ subsets. Mean (SEM) frequencies of cells within G_o_ (Ki67^−^7AAD^+^) G_1_ (Ki67^+^7AAD^−^) and S/G_2_/M (Ki67^+^7AAD^+^).

Overall, these data support that −7/del(7q) alone is an independent predictor of poor prognosis in MDS, validates that the isolated −7/del(7q) MDS cases investigated for their stem/progenitor cell hierarchies in our studies are representative for the patient group as a whole, and further establish that isolated −7/del(7q) MDS represents a high-risk MDS group distinct from −7/del(7q) cases with a complex karyotype and frequent *TP53* mutations. For the remaining part of the study we focused on analysis of the hematopoietic stem and progenitor cell compartments of MDS patients with isolated monosomy 7 (isolated −7). BM mononuclear cells from isolated −7 patients with varying blast percentages were analyzed for expression of cell surface markers used to identify normal hematopoietic stem and progenitor cell subsets [22, 23] (Figure 2A; Supplemental Figure 2). In contrast to our recent analysis of low intermediate-risk MDS patients [25], a consistently altered stem and progenitor profile was observed when comparing isolated −7 MDS cases to age-matched healthy controls (Figure 2A-2B). Independent of the BM blast percentage we observed a marked reduction, on average 66-fold (*p* = 0.001), of LIN^−^CD34^+^CD38^low/−^CD90^+^CD45RA^−^ stem cells (Figure 2B). Moreover, the LIN^−^CD34^+^CD38^low/−^ compartment was, in contrast to normal LIN^−^CD34^+^CD38^low/−^ BM cells, dominated by cells aberrantly co-expressing CD45RA (Figure 2A-2B; Supplemental Figures 2-3). Similar to the observed reduction in lympho-myeloid primed progenitors (LMPPs) with age in mice [32], the recently described human LMPP-like LIN^−^CD34^+^CD38^low/−^CD90^−^CD45RA^+^ compartment [18, 33] represented only 0.014% (± 0.006%) of total BM mononuclear cells in healthy age-matched controls. By contrast, on average a 22-fold (*p* = 0.02) expansion of this compartment was observed in isolated −7 BM (Figure 2B). Notably, in patients with higher blast counts (*n* = 3) we observed an aberrant LIN^−^CD34^+^CD38^low/−^CD90^+^CD45RA^+^ population (Supplemental Figure 2) not previously described in normal BM or cord blood [22, 34]. Moreover, CD45RA expressing cells were significantly expanded (3-fold, *p* = 0.035) within the LIN^−^CD34^+^CD38^+^CD123^+^CD45RA^+^ compartment (Figure 2B) representing granulocyte-macrophage progenitors (GMPs) in normal BM [23]. In parallel, in patients with > 10% blasts (*n* = 3), we observed a reduction of the LIN^−^CD34^+^CD38^+^CD123^+^CD45RA^−^ and LIN^−^CD34^+^CD38^+^CD123^−^CD45RA^−^ compartments, representing normal common myeloid progenitors (CMPs) and megakaryocyte and erythroid progenitors (MEPs) compartments [23], respectively, containing predominantly erythroid progenitor activity (Supplemental Figure 2). The above-described immunophenotypic features observed in Isolated −7 MDS with high blast counts were not consistently observed in a cohort of int-2/high risk IPSS MDS patients with varying risk features and high blast counts but without chromosome 7 abnormalities (Supplemental Figure 2; Supplemental Table 1).

FISH analysis of the purified stem and progenitor cell populations demonstrated high −7 involvement in all LIN^−^CD34^+^CD38^low/−^ and LIN^−^CD34^+^CD38^+^ progenitor cell compartments whereas only a very low proportion of the suppressed LIN^−^CD34^+^CD38^low/−^CD90^+^CD45RA^−^ HSC compartment detectable in two patients was part of the monosomy 7 clone (Figure 2C). By contrast, the three expanded CD45RA^+^ compartments showed the highest −7 involvement. Moreover, all the oncogenic mutations identified could also be found in all the subpopulations analysed, and typically to be highly present, with the only exception being IDH2 and JAK2 mutations that were found at lower frequencies in one of the patients (Supplemental Figure 4). Despite the disrupted stem and progenitor distribution, quiescent cells were predominantly enriched within the LIN^−^CD34^+^CD38^−^ compartment in all four cases of isolated −7 MDS which were analyzed for cell cycle status, as observed for healthy age-matched controls (Figure 2D). Due to limited cell numbers we were unable to assess monosomy 7 clonal involvement in the quiescent and proliferating fractions. However, since the CD90^−^CD45RA^+^ and/or CD90^+^CD45RA^+^ subsets represented the majority of the LIN^−^CD34^+^CD38^−^ cells in all four cases which showed very high clonal involvement (unpublished observation), the monosomy 7 clonal involvement is predicted to be high in the LIN^−^CD34^+^CD38^−^ quiescent fraction.

**Figure 3:**
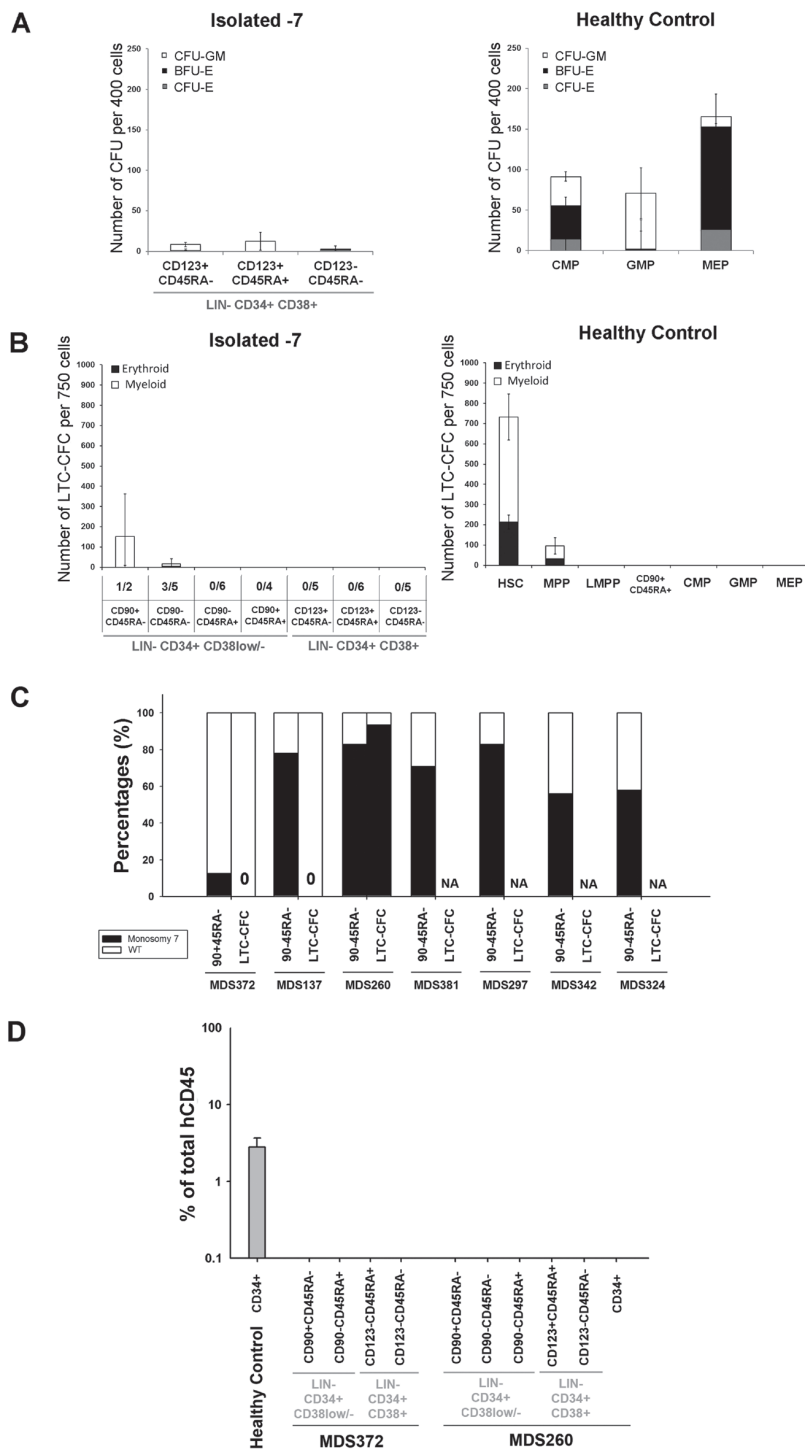
Functional assessment of stem and progenitor cells in isolated monosomy 7 MDS **A.** Mean (SEM) colony forming activity from indicated stem/progenitor cell populations from isolated −7 patients (*n* = 7) and HC (*n* = 6). Mean (SEM) burst forming unit-erythroid (BFU-E), CFU-erythroid (CFU-E) and CFU-granulocyte/monocyte (CFU-GM) based on colony morphology. **B.** Mean (SEM) LTC-CFC activity of indicated cell populations from isolated −7 (*n* = 6) and HC (*n* = 5). For isolated −7 MDS, the frequency of patients with readout is indicated below the x-axis for each population. **C.** Percentages of sorted stem cells and LTC-CFCs derived from LIN^−^CD34^+^CD38^−^CD90^+^CD45RA^−^ or LIN^−^CD34^+^CD38^low/−^CD90^−^CD45RA^−^ cells with (black) and without (white) −7 karyotype as detected by FISH. 10, 8 and 16 LTC-CFC colonies were picked and analysed for MDS372 MDS137 and MDS260, respectively. NA = Not applicable as no LTC-CFC readout **D.** Mean (SEM) human CD45^+^ engraftment in NSG mice 5 months after transplantation of CD34-enriched cells (CD34^+^) from one HC and indicated populations from 2 isolated −7 patients. Three NSG mice were used per sorted population.

Similar to what has been reported for AML [35-37] the purified stem and progenitor cell populations from isolated −7 patients showed dramatically reduced functional output in available stem and progenitor cell assays (Figure 3). Both, purified LIN^−^CD34^+^CD38^+^CD123^+^CD45RA^+^ GMPs and LIN^−^CD34^+^CD38^+^CD123^−^CD45RA^−^ MEPs showed reduced readout of myeloid and erythroid colonies, respectively (Figure 3A). Moreover, while the rare LIN^−^CD34^+^CD38^low/−^CD90^+^CD45RA^−^ cells that could be detected in one of the −7 MDS patients read out with some long-term culture activity (Figure 3B), none of these colonies carried −7 (Figure 3C), demonstrating that the LTC-CFC activity was not derived from the −7 clone. However, in one of three patients where LIN^−^CD34^+^CD38^low/−^CD90^−^CD45RA^−^ multi-potent progenitors (MPPs) cells could be analyzed, LTC-CFC proved to be part of the −7 clone (Figure 3C). In agreement with the LTC-CFC data, none of the transplanted cell populations from two patients were able to reconstitute immune-deficient mice *in vivo*, either at 5 weeks (data not shown) or 5 months (Figure 3D) after transplantation.

### Monosomy 7 clone expands within the myeloid but not erythroid lineage

**Figure 4:**
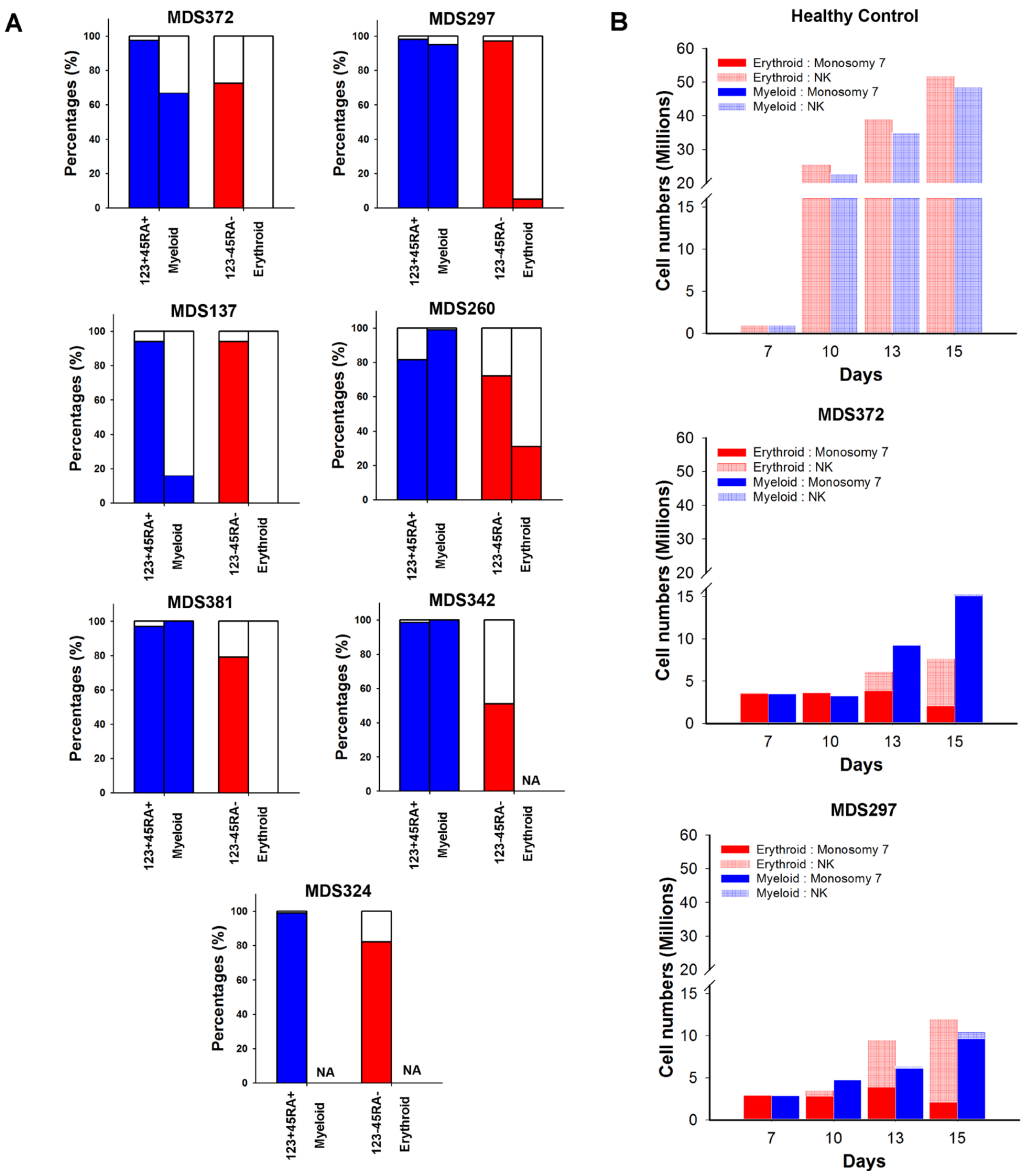
Myeloid and erythroid differentiation potential of progenitors harboring monosomy 7 **A.** Percentages of nuclei, with (colored) and without (white) −7 karyotype as identified by FISH in phenotypically defined myeloid (LIN^−^CD34^+^CD38^+^CD123^+^CD45RA^+^) or megakaryocyte-erythroid (LIN^−^CD34^+^CD38^+^CD123^−^CD45RA^−^) progenitor cells and individually picked Myeloid or Erythroid colonies, respectively, derived from these progenitor cells. A minimum of 8 colonies were analysed from each patient. NA = not applicable as no colony formation. **B.** Absolute number of cells with and without −7 karyotype as assessed by FISH ( > 300 cells counted) at indicated time points during myeloid (blue) or erythoid (red) cell suspension cultures of CD34^+^ cells. Data on y axis presented in split graphs.

The expansion of GMPs and reduction of MEPs in isolated −7 MDS indicated a preferential expansion of the myeloid in preference to the erythroid differentiation pathway already at the progenitor stage. This was further supported by a higher −7 clonal involvement in purified GMPs than MEPs in the majority of the investigated patients (Figure 4A). To investigate whether this differential impact also affected differentiation beyond the GMP and MEP stages, we picked the rare myeloid and erythroid colonies that developed from the GMPs and MEPs, respectively. In 4 of the 6 investigated patients the percentage of −7 involvement in myeloid colonies was equal or higher than in the input sorted GMP cells (Figure 4A). By contrast, the frequency of −7 erythroid colonies derived from −7 MEPs was consistently lower than the frequency of −7 cells in the input sorted CD123^−^CD45RA^−^ cells (Figure 4A). These data were further corroborated by experiments in which CD34^+^ cells were grown *in vitro* in cell suspension cultures supporting either erythroid or myeloid differentiation (*n* = 2) (Figure 4B). While the −7 clone dominated during myeloid conditions, it gradually decreased during erythroid expansion and maturation. Furthermore, compared to erythroid expansion of normal CD34^+^ cells where the culture at day 15 was dominated by CD36^+^ erythroid cells ( > 95%), corresponding cultures with monosomy 7 CD34^+^ progenitors largely generated myeloid progenitor cells (40% CD33^+^) (Supplemental Figure 5).

### Azacitidine treatment fails to efficiently target CD45RA^+^ progenitors in monosomy 7 patients

**Figure 5:**
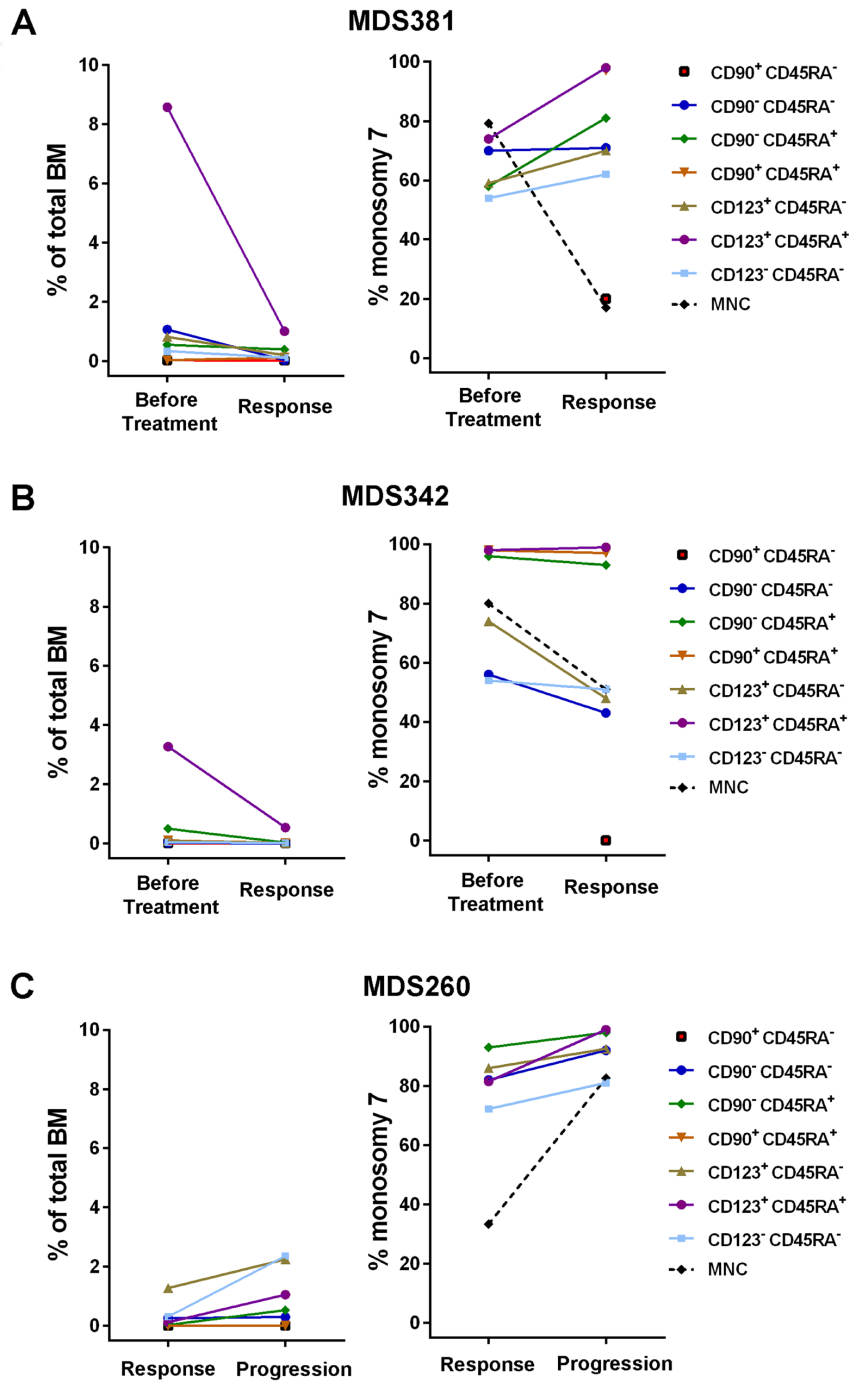
Monosomy 7 clonal involvement of stem and progenitor cell populations during azacitidine treatment Frequencies of distinct stem and progenitor cells within total BM mononuclear cells and −7 clonal involvement as determined by FISH, before and during azacitidine treatment for three isolated −7 patients. **A.** Patient MDS381 before treatment and at response after 6 cycles of azacitidine treatment. **B.** Patient MDS342 before treatment and at response after 1 cycle of azacitidine. **C.** Patient MDS260 at response after 10 cycles of azacitidine and at progression after 16 cycles of azacitidine. MNC = Mononuclear Cells.

To address if and how distinct stem and progenitor cell subsets respond to azacitidine treatment, the size and clonal involvement of stem and progenitor cell subsets was evaluated in two −7 patients, serially sampled before and during azacitidine treatment (Supplemental Table 1). Azacitidine treatment resulted in clinical improvement with a marked reduction of the −7 clone as determined by FISH in BM mononuclear cells (Figure 5A-5B). However, in all CD45RA^+^ progenitor subsets, which are typically expanded in −7 MDS patients (Figure 2A-2B), the −7 clonal involvement remained higher than in total BM and in the CD45RA^−^ progenitor subsets. In particular, the LIN^−^CD34^+^CD38^+^CD123^+^CD45RA^+^ (GMP) population, remained highly clonally involved ( > 98% −7 in both patients) after treatment.

A third isolated −7 MDS case (Figure 5C), initially showed a major response in terms of improvement in cytopenia and reduction in BM blasts to 3%, and a reduction in −7 involvement to 32%, after 10 cycles of azacitidine (Supplemental Table 1). However, the patient progressed after another 6 cycles with relapse in pancytopenia and an increase in BM blasts to 8.5% and increased −7 involvement to 82%. At both time points, stem and progenitor populations had higher −7 involvement than detected in total BM, with the −7 clonal involvement of the LIN^−^CD34^+^CD38^+^CD123^+^CD45RA^+^ (GMP) population increasing from 81% to 99% at the time of progression.

## DISCUSSION

Recent therapeutic breakthroughs in MDS have almost exclusively been associated with specific genetic characteristics in lower-risk patients [4]. Higher-risk MDS has an overall dismal prognosis unless successful stem cell transplantation can be performed [2] and the fact that almost all patients treated with azacitidine and more than a third of those receiving SCT relapse in their disease constitutes a major clinical challenge [38, 39]. One important step towards identifying improved therapeutic targets for these high-risk patients is through identification and characterization of the disease-propagating stem and progenitor cells. Although next-generation sequencing has further helped to define the genetic architecture of MDS allowing for further subdivision of previously uniformly risk-stratified monosomal karyotype patients based on detected mutations such as *TP53* [31], monosomy 7 still constitutes a poor-risk cytogenetic subgroup in both the IPSS and the IPSS-R system [2, 6, 7]. We therefore consider monosomy 7 MDS as a representative group of IPSS higher-risk MDS, even if two patients in our cohort were classified as intermediate-risk according to the IPSS-R system. We initially assessed two separate cohorts of patients with −7 or del(7q) (Karolinska Institute, Sweden; Sanger Institute, UK) for chromosome 7 clonal involvement and for co-occurrence and mutual exclusivity of other genetic events. Patients with isolated −7/del(7q) showed high clonal involvement in bulk mononuclear cells and lacked a specific pattern of co-occurring mutations. In line with a recent publication describing *TP53* mutations in patients with myeloid malignancies [40], we observed that while −7 patients with a complex karyotype frequently carried *TP53* mutations (known to signify poor prognosis [8]), this was not seen in the patients with isolated −7.

To specifically explore the disease-driving stem and progenitor cells in higher risk MDS patients, we selected patients with isolated −7 for further phenotypic and molecular characterization. In these patients we observed a dramatic reduction in cells with a LIN^−^CD34^+^CD38^−^CD90^+^CD45RA^−^ HSC phenotype and an extensive expansion and domination of highly clonally involved CD45RA^+^ GMP and LMPP-like progenitors, similar to what has been reported in AML [18], and in contrast to what has been reported in low- and intermediate-risk MDS [25]. Interestingly, expansion of GMPs has been demonstrated to be required for development of AML in mouse models [41]. Moreover, the LIN^−^CD34^+^CD38^low/−^CD90^+^CD45RA^+^ cells, which virtually does not exist in normal BM, was expanded in −7 patients with higher blast counts. Since this CD90^+^CD45RA^+^ progenitor population within the LIN^−^CD34^+^CD38^low/−^ is an aberrant cell population, it could potentially represent a highly specific biomarker for detection of the −7 clone in MRD, for instance following allogeneic stem cell transplantation. FISH analysis of the LIN^−^CD34^+^CD38^low/−^CD90^+^CD45RA^−^ subsets was as described done on 2 samples only, since the size of this subset was extremely small (less than 100 cells obtained) in the remaining samples, which in itself, constitutes an important finding, suggesting that the −7/del(7q) clone suppresses the normal LIN^−^CD34^+^CD38^low/−^CD90^+^CD45RA^−^ HSC compartment, as previously reported in AML [18].

While our findings establish a consistently distinct perturbed stem and progenitor cell hierarchy in higher risk MDS patients with monosomy 7 as the sole cytogenetic abnormality, distinct from that previously reported for lower-risk MDS patients [25], it remains to be investigated to what degree this phenotype is common for high-risk MDS regardless of chromosome 7 abnormalities. Although we did not observe the same phenotypic changes in 6 cases of int-2/high-risk non −7 MDS cases included in our analysis, this might reflect the limited number and heterogenous nature of the patients investigated. In fact, the finding of qualitatively similar although quantitatively more extensive stem/progenitor perturbations in AML [18], suggests that our findings are likely to be a common feature in additional groups of higher risk MDS. Therefore, it will be important to investigate to what degree these findings in −7 MDS patients can be extrapolated to additional groups of high-risk MDS.

While another study [24] implicated an expansion rather than reduction of LIN^−^CD34^+^CD38^low/−^CD90^+^ HSCs in higher-risk MDS, the CD45RA status in the LIN^−^CD34^+^CD38^low/−^CD90^+^ compartment did not appear to have been investigated, of considerable significance since our studies show that the vast majority of LIN^−^CD34^+^CD38^low/−^CD90^+^ BM cells in isolated Monosomy 7 patients aberrantly express CD45RA, and to be highly clonally involved, at distinction of the normal HSC compartment which is CD45RA^−^ [22].

Our cell cycle analysis in −7 MDS stem and progenitor cells is difficult to interpret, as it was performed only within the two major CD34^+^CD38^−^ (stem/primitive progenitor-containing) and CD34^+^CD38^+^ (progenitor-containing) populations, and these represent largely different stem/progenitor populations in normal and monosomy 7 bone marrow. Nevertheless, we observed a clear distinction between CD34^+^CD38^−^ cells being predominantly in G0 (quiescence) and CD34^+^CD38^+^ cells predominantly in G1, of relevance for the challenge of targeting quiescent cells with many therapeutic drugs.

In our isolated −7 MDS cohort, a severely compromised LIN^−^CD34^+^CD38^low/−^CD90^+^CD45RA^−^ HSC compartment was accompanied by reduced normal stem cell activity as analyzed by *in vitro* LTC-CFC, and xenotransplantation assays. Since the HSC compartment predominantly was not part of the −7 clone, our findings are most compatible with the −7 clone extrinsically suppressing the normal HSC compartment. Although only representing a limited number of patients in our study, the inability of all −7 stem and progenitor cell populations from most of the investigated −7 high-risk MDS patients, to read out in available *in vitro* and *in vivo* stem cell assays is reminiscent of what has been reported for a high fraction of patients with AML [36, 37], most frequently in patients with relatively better prognosis than those that frequently engraft in immune-deficient mice [35, 42]. While this obviously represents a considerable limitation towards identification of the CSCs which must be responsible for propagating the malignant clone(s) in high-risk MDS and AML patients [12, 14], it is noteworthy that this similar observation in isolated −7 MDS patients and AML, is paralleled by a stem and progenitor phenotype which is also very similar to that frequently found in AML patients [18], and distinct from reported low-risk MDS patients. Thus, when compared to low-risk MDS, −7 MDS patients which have a high-risk of transforming to AML, have a stem and progenitor phenotype which already much more resembles that of AML.

Myelo-erythroid differentiation, as assessed by clonogenic potential, was severely reduced, but with a distinct and consistent impact on development of the myeloid and erythroid lineages. The −7 clone was dominating in the myeloid (GM) lineage, as demonstrated by very high clonal involvement in GMPs as well as in further expanded myeloid progeny. By contrast we observed a selective defect at multiple steps in erythroid development, as erythroid progenitors consistently showed lower −7 involvement than GMPs, and also in the further expansion process from erythroid progenitors, the −7 clone had a distinct disadvantage. The differential impact on myeloid and erythroid differentiation might reflect effects on differentiation, cell cycle and/or apoptosis.

Interestingly, *EZH2*, located within the commonly deleted region (CDR) of 7q [43], has been implicated to play an important role in erythropoiesis, since *Ezh2*-deficient embryos have defective erythropoiesis [44]. Furthermore, reduced expression of *DOCK4* in hematopoietic progenitors, another gene within the 7q CDR, was recently shown to result in dysregulated erythroid development [45]. As previously suggested, more than one haploinsufficient gene might co-operate in producing the hematopoietic phenotype in −7/del(7q) patients [43].

Since most if not all isolated monosomy 7 patients are likely to also have other recurrent driver mutations, as confirmed here, it remains to be established through genetic modeling studies, to what degree the perturbed stem and progenitor hierarchies and differentiation defects identified here reflect an impact of the chromosome 7 deletion itself, and/or other co-occurring recurrent genomic lesions. Again, the finding of a similar stem-progenitor perturbation in AML, regardless of having chromosomal 7 abnormalities or not, suggest that these perturbations might be caused by a number of recurrent genomic lesions, potentially including monosomy 7.

Finally, we assessed how azacitidine, an inhibitor of the DNA methylotransferase activity and a first-line treatment, for higher-risk MDS, affects the −7 stem and progenitor cell compartments. The impact of azacitidine, in combination with other drugs, on leukemic stem/progenitor cells of high-risk AML and MDS patients has previously been reported, and is associated with a failure to effectively eradicate distinct stem and progenitor cell populations [24, 46]. Herein, we similarly demonstrate that while azacitidine treatment in the investigated isolated −7 MDS patients reduced the MDS clone size in total BM mononuclear cells in parallel with a clinical response, the −7 clone remained stable or even expanded within distinct progenitor subsets and in particular in the CD45RA^+^ subsets, including GMP and LMPP-like subsets. These findings are supported by a similar case-report in another study [24], although it will be important to extend these observations to a larger patient cohort.

In conclusion, we provide evidence that patients with isolated −7 high-risk MDS have a perturbed stem and progenitor profile with a much closer resemblance to that reported in de novo AML than low- to intermediate- risk MDS. Most notably, the progenitor compartment is dominated by distinct CD45RA^+^ progenitor subsets which appear to be highly therapy-resistant. Further molecular characterization of these perturbed progenitors subsets should help identify novel and improved therapeutic targets to more effectively eliminate −7 stem and progenitor cells.

## MATERIALS AND METHODS

### Patient cohorts

All patients were classified according to WHO 2008 Classification and risk-classified according to the IPSS and IPSS-R Prognostic Scoring System. MDS patients were enrolled at the Karolinska University Hospital in Stockholm, Sweden (MDS coded patients; *n* = 21, where 13 were DNA sequenced) and grouped based on their cytogenetic aberrations (Supplemental Table 1). The patient cohort from the Sanger Institute in Cambridge, United Kingdom (PD coded patients; *n* = 22, where 14 were DNA sequenced) was used as a meta-analysis cohort of patients from previously published data [11]. MDS patients with isolated −7 were investigated regardless of the blast percentages and information with regard to blast percentages of individual patients is included in Supplementary Table 1 along with other clinical characteristics. The patient study, as well as the analysis of healthy bone marrow (BM) samples was approved by ethical committees for clinical research both in Sweden and the United Kingdom.

### Targeted DNA sequencing

Samples for targeted genomic enrichment and sequencing by Haloplex selector probes were prepared according to manufacturer's instructions (Agilent) and as previously described [25]. Briefly, genomic DNA was isolated from unfractionated BM cells by a DNA isolation kit (Sigma), followed by enzymatic digestion and validation using 2200 TapeStation High Sensitivity D1K assay (Agilent). The genomic DNA restriction fragments were hybridized to Haloplex probes targeting regions of interest. Detailed methods for the identification of mutations and computational analysis predicting order of events are described in Supplemental Methods.

### Flow cytometry and fluoresence-activated cell sorting (FACS)

Stem and progenitor cell analysis and purification from BM mononuclear cells was performed as described in Supplemental Methods.

### Functional *in vitro* and *in vivo* assays for stem and progenitor cell activity

Detailed methods for colony forming unit (CFU), long-term culture colony forming cell (LTC-CFC), erythroid and myeloid cell suspension cultures and *in vivo* xenograft transplantation are described in Supplemental Methods.

### Fluorescence *in situ* hybridization (FISH)

Cells, FACS sorted directly onto glass slides, or picked CFU and LTC-CFC colonies were used for cytogenetic analysis by interphase FISH using Poseidon FISH DNA probe on MDS del(7q) (7q22, 7q36)/SE 7 Triple color (Kreatech) according to manufacturer's instructions. Fluorescence images were obtained using fluorescence microscopy (Olympus; model BX60F-3). The probe hybridizes to 7q22 (green), 7q35 (red) and chromosome 7 centromere (blue). Nuclei with 2 green, 2 red and 2 blue signals were considered as normal karyotype (NK), whereas nuclei where at least one loci was detected as single color were considered to contain a partial or complete deletion of chromosome 7 (−7/del(7q)). Unless otherwise specified, a minimum of 100 nuclei were analyzed per sorted cell population and 50 nuclei per picked colony. Since picked colonies might contain contaminating cells from other progenitors, if 80-100% of nuclei were −7 the colonies were classified as −7, if 0-20% of nuclei were −7 as normal karyotype, and as inconclusive if 21-79% of nuclei were −7.

### Statistical analysis

Survival plots were generated using Sigma Plot 12.0 software and Graph Pad Prism. For individual comparisons, a non-parametric Mann-Whitney statistical test was used, and a p-value less than 0.05 considered significant.

## SUPPLEMENTARY MATERIALS FIGURES AND TABLES


