# Supplementary material for: Perturbed hematopoietic stem and progenitor cell hierarchy in myelodysplastic syndromes patients with monosomy 7 as the sole cytogenetic abnormality
Source: Oncotarget. 2016 Sep 24;7(45):72685–98. doi: 10.18632/oncotarget.12234 (PMC5341937; doi:10.18632/oncotarget.12234)
Supplement: Supplementary file 1 [file oncotarget-07-72685-s001.pdf]

# Perturbed hematopoietic stem and progenitor cell hierarchy in myelodysplastic syndromes patients with monosomy 7 as the sole cytogenetic abnormality

## Supplementary Material

### Targeted DNA sequencing for identification of mutations

Two datasets were included in order to specifically investigate the genetic architecture of MDS patients with a chromosome 7 aberration including monosomy 7 or del(7q) as isolated or complex karyotype: 1) Targeted sequencing of 42 genes (listed in table below) recurrently mutated in myeloid malignancies by HaloPlex (n = 13), and 2) Meta-analysis of a previously reported study [1], where MDS patient containing loss of chromosome 7 were selected for detailed analysis (n = 14).

| Gene ID | Gene name |
|---|---|
| ASXL1 | additional sex combs like 1 (Drosophila) |
| BCOR | BCL6 corepressor |
| CBL | Cbl proto-oncogene, E3 ubiquitin protein ligase |
| CEBPA | CCAAT/enhancer binding protein (C/EBP), alpha |
| CSF3R | colony stimulating factor 3 receptor (granulocyte) |
| DNMT3A | DNA (cytosine-5-)-methyltransferase 3 alpha |
| EPOR | erythropoietin receptor |
| ETV6 | ets variant 6 |
| EZH2 | enhancer of zeste homolog 2 (Drosophila) |
| FLT3 | fms-related tyrosine kinase 3 |
| GATA1 | GATA binding protein 1 (globin transcription factor 1) |
| GATA2 | GATA binding protein 2 |
| GATA3 | GATA binding protein |
| IDH1 | isocitrate dehydrogenase 1 (NADP+), soluble |
| IDH2 | isocitrate dehydrogenase 2 (NADP+), mitochondrial |
| JAK2 | Janus kinase 2 |
| KDM6A | lysine (K)-specific demethylase 6A |
| KIT | v-kit Hardy-Zuckerman 4 feline sarcoma viral oncogene homolog |
| KRAS | v-Ki-ras2 Kirsten rat sarcoma viral oncogene homolog |
| MLL | myeloid/lymphoid or mixed-lineage leukemia (trithorax homolog, Drosophila) |
| MPL | myeloproliferative leukemia virus oncogene |
| NPM1 | nucleophosmin (nucleolar phosphoprotein B23, numatrin) |
| NRAS | neuroblastoma RAS viral (v-ras) oncogene homolog |
| PDS5B | regulator of cohesion maintenance, homolog B (S. cerevisiae) |

| | |
|---|---|
| PRPF40B | PRP40 pre-mRNA processing factor 40 homolog B (S. cerevisiae) |
| RAD21 | RAD21 homolog (S. pombe) |
| RUNX1 | runt-related transcription factor 1 |
| SF1 | splicing factor 1 |
| SF3A1 | splicing factor 3a, subunit 1, 120kDa |
| SF3B1 | splicing factor 3b, subunit 1, 155kDa |
| SH2B3 | SH2B adaptor protein 3 |
| SMC1A | structural maintenance of chromosomes 1A |
| SMC3 | structural maintenance of chromosomes 3 |
| SRSF2 | serine/arginine-rich splicing factor 2 |
| STAG1 | stromal antigen 1 |
| STAG2 | stromal antigen 2 |
| TET2 | tet methylcytosine dioxygenase 2 |
| TP53 | tumor protein p53 |
| U2AF1 | U2 small nuclear RNA auxiliary factor 1 |
| U2AF2 | U2 small nuclear RNA auxiliary factor 2 |
| WT1 | Wilms tumor 1 |
| ZRSR2 | zinc finger (CCCH type), RNA-binding motif and serine/arginine rich 2 |

HaloPlex prepared library were sequenced on an Illumina HiSeq and the sequencing data were aligned and processed as follows: 1) Adapter sequences were removed using CutAdapt with the following adaptor sequence input: GATCGGAAGAGCACACGTCTGAACTCCAGTCAC. 2) The raw sequences were aligned to the Hg19 build using the Mosaik aligner [2]. Minimum alignment percentage was set to 0.95 and percentage of mismatches allowed to 0.05. 3) The reads were amplicon mapped with a modified version of GATK-lit [3]. 4) Positions with base quality ≥ 20, mapping quality ≥ 5, read depth ≥ 20 and variant allele ratio ≥ 1% were used for further analysis, 5) Variants were annotated with Annovar (2013-08-23 release).

We identified and selected candidate mutations by excluding 1) variants in splicing or intronic regions, 2) synonymous changes, 3) variants present >65 times per 96-plexed plate (redundant variants).

A cut-off of variant read frequency ≥5% with ≥20 variant allele reads was used. SNPs present >0.0014 in 1000 genome project human SNP database and low quality reads with multiple mismatches (based on visual inspection of the raw and aligned data) were also excluded.

In absence of available somatic control DNA, mutations were grouped as: 1) “Oncogenic”, known oncogenic variants previously reported in the literature as previously listed in hematopoietic and lymphoid malignancies in the COSMIC database (version 70), 2) “Truncating”, nonsense mutations or frameshift insertions or deletions and 3) “Unknown”, predicted as “DAMAGING” single amino acid changes by SIFT [4] in genes implicated in myeloid malignancies.

The identification of mutations in patients analyzed by meta-analysis has been described previously [1] where COSMIC database, version 60, was used.

**Computational analysis from Targeting Sequencing data.**

In order to predict the order of acquisition of chromosome 7 loss and mutations in our selected -7/del(7q) patient cohort the following strategy was used:

1) Initially we identified copy number aberrations (CNAs), if any, in the region of the mutations, since calculation of fraction of cells having a mutation is affected by the presence of copy number changes. Allele frequencies of reference (REF) and alternate (ALT) alleles of all SNPs within the 1000 genomes dataset [5] lying on the chromosome bearing the mutation were obtained. Visual inspection of plots of beta allelic frequency (BAF) and normalised coverage (logR) identified samples subject to CNAs in the region of the mutation. Copy-neutral loss of heterozygosity (LOH) was identified in *JAK2*, *TP53* and *DNMT3A* in several samples while mutations on chromosome 7 were assumed to be in regions of copy number loss. Gains were identified in the region of *RUNX1* in PD7037a.

2) For chromosome 7 regions and regions with other CNAs detected, informative heterozygous SNPs were identified as those loci with BAF between 0.01 and 0.99 (inclusive), a minimum of 5 reads excluding SNPs present at a frequency < 0.0014 in 1000 genome project human SNP database. For patients sequenced by HaloPlex, this analysis was restricted

to 2-4 chromosome 7 SNPs identified within the *EZH2* gene locus, whereas for the meta-analyzed patients the SNPs spanned various regions within chromosome 7 (*ATXN7L1* - 7q22.3, *BRAF* - 7q34, *CUX1* – 7q22.1, *EGFR* – 7p12, *EZH2* – 7q35-36, *HIPK2* – 7q34, *IKZF1* – 7p13, *MET* – 7q31, *MLL3* – 7q36.1, *MLL5* – 7q22.3, *MMD2* – 7p22.1, *RINT1* - 7q22.3 and *SRPK2* – 7q22.3).

3) For each informative heterozygous SNP, bootstrap resampling of REF and ALT reads was performed (n = 10000) and the fraction of cells bearing a copy number change, *f*, was calculated using a previously derived equation [6]

$$f / (fn_t + (1-f)n_n) = m / n_{mut}$$

where $n_t$ and $n_n$ are, respectively, the locus-specific copy number in the tumour cells and the locus-specific copy number in the normal cells, $n_{mut}$ is the number of chromosome copies bearing the mutation and m is the b-allele frequency.

4) Using the same bootstrap resampling technique, the number of reads reporting a mutation was resampled from the observed data and the equation above used to convert the BAF of the mutation to the fraction of cells bearing the mutation. Differences in fraction of cells bearing each mutation were assessed at a significance level of 0.01, i.e. they were significant only if 99% of the bootstrap samples reported the same ordering.

The computational calculation of fraction of cells with -7 was validated by comparing with FISH data gathered from the same sampling time-point for 3 patients shown in the table below.

| Patient ID | Population | FISH (% of -7 cells) | Computational (% fraction of -7 cells) |
|---|---|---|---|
| MDS381 | BM mononuclear cells | 82 | 93 |
| MDS342 | BM mononuclear cells | 81 | 84 |
| MDS260 | BM mononuclear cells | 70 | 82 |

## Sanger Sequencing

All non-recurrent mutations (truncating and unknown) detected by HaloPlex were independently validated by Sanger Sequencing. DNA from BM mononuclear cells from patients was amplified with specific primers designed for 400 to 600 base pair amplicons spanning the nucleotide base of interest and optimal annealing Tm 57°C with Primer 3 Input software (primers used in table below). The PCR products were purified by enzymatic digestion of dNTPs and primers with Exosap-IT (Affymetrix) according to manufacturer's instructions. Sequencing data were analysed using ApE software.

| | | **PRIMERS (5' - 3')** |
|---|---|---|
| **ASXL1_Y591X** | Forward | GAGCAGAATCTTCTCTGAATGGTG |
| | Reverse | CCATCTGTGAGTCCAACTGTAGC |
| **ETV6_L117F** | Forward | TCGCTCTGACCTTTTGAGTT |
| | Reverse | CTTCGCTTTGATCGTGAGC |
| **ETV6_D372V** | Forward | ATCAGTCAACCCAAGCTAGG |
| | Reverse | CCAGCAAACCAGGATATGTG |
| **DNMT3A_L500fs** | Forward | TGTCATCAGGACTCTGCTTCCAG |
| | Reverse | TTCTGTCCTGCTGTCACTCCATC |
| **SH2B3_V402M** | Forward | CTTATCTAACAGGTGCTTCTCCTG |
| | Reverse | ACGACTACCACGTAGCTGGAGAG |
| **ASXL1_E773X** | Forward | GGAAAGTGTACGTCAGATCTACAG |
| | Reverse | GTGAGGATTCAGGTGTGGAACTG |
| **EZH2_H655R** | Forward | CTAATGCTCATGGCAAAGTGACC |
| | Reverse | CAAACCCTGAAGAACTGTAACCAG |
| **KMD6A_N777K** | Forward | GGTGGACAACAAGGCATTAC |
| | Reverse | TTCATGGGGCTCTGAGATTC |
| **ASXL1_G645fs** | Forward | GAGCAGAATCTTCTCTGAATGGTG |
| | Reverse | CCATCTGTGAGTCCAACTGTAGC |
| **TET2_K111X** | Forward | TCCCATTTGCCAGACAGAAC |
| | Reverse | CTTTTCCCCTCCTGCTCATT |
| **RUNX1_P103S** | Forward | AGAAAGCTGAGACGAGTGCCTC |
| | Reverse | GTTTGCAGGGTCCTAACTCAATC |
| **IDH1_N171fs** | Forward | TTCTGCCAACCTTCTCTCTGTGC |
| | Reverse | GGAATGTCTGGACCTCTTCATCC |

| | | |
|---|---|---|
| **EZH2_G151X** | Forward | CAAGTGCTAGGATTACAGAGTTAG |
| | Reverse | TTTGCTTCCTTTGCCTAACACCAG |
| **BCOR_R1417W** | Forward | CTACCTGCCTGAACACGAACTG |
| | Reverse | CTTAACCGTGTGCGCTGCTTG |
| **RUNX1_S266fs** | Forward | GCCTTCACATCCACAATCTCTGC |
| | Reverse | CAACAGAGGCAGATACTTGGAC |
| **RUNX1_A217fs** | Forward | CCAAGACTCTGCATTTCCAACAGC |
| | Reverse | AGCTGGCATATCTCTAGCGAGTC |

**Flow cytometry and fuoresence-activated cell sorting (FACS)**

BM mononuclear cells and $CD34^+$ cells were prepared for flow cytometry analysis and sorting as previously described [7]. Briefly, BM mononuclear cells were isolated by Lymphoprep® (Fresenius Kabi Norge AS) gradient centrifugation and $CD34^+$ cells were enriched using a magnetic activated cell sorting (MACS) isolation kit (Miltenyi Biotec). Viable BM mononuclear cells and $CD34^+$ cells were cryopreserved in Iscoves Modified Dulbecco Medium (IMDM, Gibco) supplemented with 50% fetal calf serum (FCS, Gibco) and 10% Dimethylsulfoxid (DMSO, Sigma Aldrich). Mononuclear cells or $CD34^+$ enriched cells were thawed in IMDM medium containing 20% FCS and 100µg/ml DNAse type II from bovine pancreas (Sigma Aldrich), washed in Dulbecco´s phosphate buffered saline (PBS, Gibco) with 5% FCS and prepared for flow cytometry staining. The cells were incubated with Fc-Blocking reagent (Miltenyi Biotec) for 10 minutes and followed by 30 minutes incubation at $4^oC$ with antibodies, as described in table below. Live cells were identified by 7-Aminoactinomycin D (7AAD, eBiosciences) exclusion. For all experiments where frequency of populations within BM were calculated or cells were sorted, both fluorescent-minus-one (FMO) and single-stained controls were used [8], except 2/6 cases from int-2 and high risk MDS cohort where only single-stain controls were used. For Figure 2D isotype control was used for Ki67. Cells were sorted and analyzed based on their immuno-phenotypic characteristics described in table below. Cell purity post-sorting was analysed and was found

to be >98% for all sorted populations. FACS ARIA or FORTESSA (Becton Dickinson) instruments used at Karolinska Institute (WIRM flow cytometry facility) and University of Oxford.

Cell cycle analysis was performed by first staining for cell surface markers, followed by fixation and permeabilizaton using Fixation/Permeabilization Solution (BD Bioscience) according to manufacturer instructions, then wash in Perm/Wash Solution (BD Bioscience) and intracellular Ki67 staining in Perm/Wash buffer for 30 minutes at room temperature. DNA was stained by 0.25 μg 7AAD (eBiosciences) per million of cells.

Analysis of cell suspension cultures was performed after 15 days of culture. Cells were washed with PBS and stained with a combination of erythroid and myeloid markers to assess differentiation efficiency. The antibodies used were CD66b-FITC (G10F5/BD Biosciences), CD33-APC (WM53/eBiosciences), CD235a-e450 (6A7M/eBiosciences), CD36-APCe780 (5-271/Biolegend), CD34-PECy7 (581/BD Biosciences) and 7-Aminoactinomycin (7AAD) for dead cell exclusion.

**FACS panels table**

| Laser | Low Pass (nm) | Filter (nm) | FACS Sorting | Engraftment and lineage distribution NSG | Cell Cycle |
|---|---|---|---|---|---|
| Blue 488 nm | 505 | 530/50 (FITC) | | **Human CD15 (MMA)/ CD33 (P67.6)/ CD66b (G10F5) (BD Bioscience)** | **Ki67 (51-36524X/ BD Bioscience)** |
| | 685 | 710/50 | | | |
| Red 633 nm (Aria) | | 670/14 (APC) | **Human CD34 (581/ Biolegend)** | **Human CD34 (581/ Biolegend)** | **Human CD34 (581/ Biolegend)** |

| | | | | | |
|---|---|---|---|---|---|
| or 640nm (Fortessa) | 690 | 730/45 (A700) | **Human CD90 (5E10/ Biolegend)** | **Human CD45 (HI30/ Biolegend)** | |
| | 750 | 780/60 | | | |
| Violet 405 nm | | 450/50 (e450/DAPI) | **Human CD38 (HB7/ eBiosciences)** | **DAPI** | **Human CD38 (HB7/ eBiosciences)** |
| | 505 | 525/50 (V500/PO) | ***LIN Biotin/ Streptavidin V500** | **Mouse CD45 (30-F11/ Biolegend)** | ***LIN Biotin/ Streptavidin V500** |
| | 600 | 610/20 (e605NC) | **Human CD45RA (HI100/ eBiosciences)** | | |
| | 630 | 670/30 | | | |
| Yellow/Green 561 nm | | 586/15 (PE) | **Human CD19 (HIB19/ eBiosciences)** | **Human CD19 (HIB19/ eBiosciences)** | **Human CD90 (5E10/ Biolegend)** |
| | 600 | 610/20 | | | |
| | 635 | 670/30 (7AAD) | **7AAD (eBiosciences)** | | **7AAD (eBiosciences)** |
| | 685 | 710/50 | | | |
| | 750 | 780/60 (PECy7) | **Human CD123 (6H6/ Biolegend)** | | |

*For LIN Biotin Cocktail: CD2-Biotin (RPA-2.10/BD Biosciences), CD3-Biotin (UCHT1/BD Biosciences), CD4-Biotin (RPA-T4/BD Biosciences), CD8-Biotin (RPA-T8/BD Biosciences), CD11b-Biotin (ICRF44/BD Pharmigen), CD14-Biotin (M5E2/Biolegend),

CD56-Biotin (B159/BD Biosciences), CD235a-Biotin (HIR2-eBiosciences), Streptavidin V500 (BD Biosciences).

**Immunophenotyping strategy table**

| Population | Abbreviations | Definition |
|---|---|---|
| HSC | Hematopoietic Stem Cells | $7AAD^{-}LIN^{-}CD34^{+}CD38^{low/-}CD90^{+}CD45RA^{-}$ |
| MPP | Multi Potent Progenitors | $7AAD^{-}LIN^{-}CD34^{+}CD38^{low/-}CD90^{-}CD45RA^{-}$ |
| LMPP-like | Lympho-Myeloid Primed Progenitors -like | $7AAD^{-}LIN^{-}CD34^{+}CD38^{low/-}CD90^{-}CD45RA^{+}$ |
| CMP | Common Myeloid Progenitors | $7AAD^{-}LIN^{-}CD34^{+}CD38^{+}CD90^{-}CD123^{+}CD45RA^{-}$ |
| GMP | Granulocyte Macrophage Progenitors | $7AAD^{-}LIN^{-}CD34^{+}CD38^{+}CD90^{-}CD123^{+}CD45RA^{+}$ |
| MEP | Megakaryocyte Erythroid Progenitors | $7AAD^{-}LIN^{-}CD34^{+}CD38^{+}CD90^{-}CD123^{-}CD45RA^{-}$ |
| $LIN^{-}$ | Lineage negative | $CD3^{-}CD4^{-}CD8^{-}CD11b^{-}CD14^{-}CD19^{-}CD56^{-}CD235ab^{-}$ |

**Colony Forming Unit (CFU) assays**.

As previously described [7]**,** 400 cells were plated in replicate plates into H4434 methylcellulose according to manufacturer instructions (Stem Cell Technologies) and colonies scored after 14 days of culture under an inverted microscope. After counting the number of colonies, single colonies were picked and cytospun for FISH analysis.

**Long-term culture colony forming cell (LTC-CFC) assay.**

Purified cells (400-2000 cells) were co-cultured with irradiated M2-10B4 and SL/SL as stromal feeder cells [9] in H5100 MyeloCult medium (StemCell Technologies) supplemented with $10^{-6}$ M hydrocortisone 21-hemisuccinate (StemCell Technologies), with weekly half-medium changes as previously described [7]. Two or three replicate wells were used for each population and individual wells were harvested after 6 weeks of culture and transferred to H4434 methylcellulose (Stem Cell Technologies). Colonies were scored under an inverted microscope and single colonies were picked for FISH analysis after 14 days of culture.

**Erythroid and myeloid cell suspension cultures.**

$CD34^+$ cells (>95% purity) were cultured ($0.1x10^6$/ml) for 14 days in Iscove's Modified Dulbecco medium (IMDM with GlutaMAX, Gibco) supplemented with 15% BIT 9500 serum substitute (Stem Cell Technology) recombinant human interleukin rh-IL-3 (10 ng/ml, Life Technologies), rh-IL-6 (10 ng/ml, Life Technologies), rh-stem cell factor (rh-SCF, 25 ng/ml, Biosource, Camarillo, CA, USA). At day 7 of culture, cells were equally distributed to an erythroid and myeloid differentiation culture conditions. For erythroid differentiation cells harvested in medium the same medium as above supplemented with Epo (2U/ml, NeoRecormon Roche, Sweden) as previously described [10, 11]. For myeloid differentiation, cells were washed and resuspended in Myelocult H5100 medium supplemented with $10^{-6}$ M hydrocortisone (StemCell Technology) and G-CSF (20ng/ml, PeproTech). Erythroid and myeloid differentiation was quantified by flow cytometric staining (Supplemental Figure 5) and confirmed by May-Grunwald Giemsa staining of cytospun cells.

**Mice and NSG transplantation experiment.**

NOD.Cg-Prkdc$^{scid}$ Il2rg$^{tm1Wjl}$/SzJ (NSG) mice, 10-14 weeks of age, were given sublethal irradiation by exposure of two doses of 1.25 Gy (Cs source) four hours apart as previously described [12]. Cells were delivered intra-femorally within 24 hours of the last irradiation dose. The cell populations were injected according to their ratios within the patient's BM. A range of 90 to 920 purified stem cells (LIN$^-$CD34$^+$CD38$^{low/-}$CD90$^+$CD45RA$^-$) and a range of 1.350 to 50.000 progenitor cells or CD34$^+$ separated cells were injected per mouse. For all the different sorted populations the above number of cells was injected in three individual NSG mice except CD34$^+$ cells from healthy control which were injected in 5 individual mice. Human engraftment and lineage distribution within NSG BM was monitored by flow cytometry after 5 weeks by BM aspiration and after 20 weeks by BM harvest from terminated mice. Human engraftment was calculated based on the frequency of human CD45 from the sum of human and mouse CD45 within the total BM. Reconstitution analysis in non-transplanted NSG mice were used to establish the minimum threshold for human CD45 staining to 0.1%. All mice were bred and maintained at the Oxford Biomedical Services and all experiments were done with the approval of the UK Home Office.

**Targeted screening of recurrent mutations on isolated stem and progenitor cells using Fluidigm SNP genotyping assays.**

To track the selected oncogenic mutations back to purified cell populations, 1.000 to 20.000 FACS purified cells were subjected to whole genome amplification by GenomiPhi V2 DNA amplification kit (GE Healthcare) according to manufacturer's instructions. Non-amplified genomic DNA (10ng) from bulk BM mononuclear cells and autologous T cells, and GenomiPhi-amplified DNA (10ng) from purified cell populations was utilized for the Fluidigm SNP Type Assays (Fluidigm Corporation). Mutation specific primers and probes

were designed by Fluidigm Corporation (see table below for sequences) and mixed with a PCR reaction mix (FastStart High Fidelity PCR System, dNTPack (Roche)) and DNA in a Fluidigm 48.48 Access Array Integrated Fluidics Circuit (IFC) using the Pre-PCR IFC Controller AX (Fluidigm), following the manufacturer's instructions. Validation analysis performed on two different assays (SRSF2_P95H and U2AF1_Q157P) and total BM mononuclear cells and autologous T cells for somatic control, either GenomiPhi-amplified or not, confirmed the performance of the method. The sensitivity of the assay was validated by serial dilution spiked-in samples of patients BM mononuclear cells, with the above two mutations, mixed together with healthy BM mononuclear cells. Our validation experiment, with the spiked-in samples, revealed that for less than 10% of cells containing a mutation the Fluidigm SNP genotyping assay could not confidentially predict the presence or absence of a mutation.

**SNP Type assay Primers**

| SNP_NAME | ASP1_NAME | ASP2_NAME | ASP1_SEQ | ASP2_SEQ | LSP_SEQ | STA_SEQ |
|---|---|---|---|---|---|---|
| **ASXL1-Y591X** | C | G | TGGTTAAAGGTCAGCCCACTTAC | TGGTTAAAGGTCAGCCCACTTAG | CGGCAGGAGGACTCCGT | CACGTATCAAACCACCCTGG |
| **IDH2-R140Q** | C | T | GAAAAGTCCCAATGGAACTATCCG | GGAAAAGTCCCAATGGAACTATCCA | CTCCCGGAAGACAGTCCCC | CAGAGTTCAAGCTGAAGAAGATGT |
| **JAK2-V617F** | G | T | TTTTACTTACTCTCGTCTCCACAGAC | GTTTTACTTACTCTCGTCTCCACAGAA | GCAAGCTTTCTCACAAGCATTTGGT | GAAAGGCATTAGAAAGCCTGTAGT |
| **SRSF2 - P95H** | C | A | CGCTACGGCCGCCC | CGCTACGGCCGCCA | GCCCCCGTACCTGCG | TGCGGGTGCAAATGGC |
| **U2AF1-Q157P** | A | C | CTCACTCACCCCATCTCATACT | CTCACTCACCCCATCTCATACG | TGTCACCCGTGACGGACTT | TCACGTCACTGGCCACTC |

# SUPPLEMENTAL TABLES

## Supplemental Table 1. Clinical information for patients from Karolinska Institute cohort.

### Complex -7

| *Patient ID* | *Visit* | *Sex* | *Age* | *WHO* | *IPSS* | *Cytogenetics* | *BM Blast (%)* | *BM Cellularity (%)* | *Hb (g/L)* | *WBC ($10^9$/L)* | *ANC ($10^9$/L)* | *Platelets ($10^9$/L)* | *Treatment* | *Included in Immunophenotyping experiments* | *Included in computational analysis* | *Included in survival analysis* | *Included in cell cycle analysis* |
|---|---|---|---|---|---|---|---|---|---|---|---|---|---|---|---|---|---|
| MDS363 | 01 | M | 70 | RAEB-2 | HIGH | 45,XY,-7,del(20)(q11.2)[21]/46,XY[4] | 11.0 | 20 | 92 | 1.8 | 0.1 | 64 | No | No | No | Yes | No |
| MDS312 | 01 | F | 83 | RAEB-1 | INT-2 | 46,XX,del(5)(q13q33)[15]/45,idem, -7(2)/46,XX[5] | 6.0 | 30 | 87 | 2.9 | 1.1 | 221 | No | No | No | Yes | No |
| MDS278 | 02 | F | 41 | RAEB-1 | HIGH | 47,XX,-7,+21,+mar[20] / 47,XX, -7,+21+22[4] / 46,XX[1] | 5.5 | 80 | 89 | 2.0 | 0.9 | 31 | No | No | No | Yes | No |
| MDS378 | 01 | M | 75 | RCMD | INT-2 | 45,XY,-7,del(11)(q13)[10]/46,XY,idem,+mar[4]/ 47,XY,idem,+8,+mar[2]/46,XY[9] | 4.5 | 50 | 101 | 2.3 | 0.8 | 55 | No | No | No | Yes | No |
| MDS265 | 01 | M | 81 | RCMD-RS | INT-2 | 44,XY,-6,-7,add(12)(q24),add(12)(q24),-15,add(19)(p13),add(20)(q13),+mar1[4]/44,idem,-13,-14,+mar2,+mar3[5]/46,XY[14] | 3.5 | 80 | 109 | 3.5 | 2.8 | 86 | No | No | No | Yes | No |

### Isolated -7

| *Patient ID* | *Visit* | *Sex* | *Age* | *WHO* | *IPSS* | *Cytogenetics* | *BM Blast (%)* | *BM Cellularity (%)* | *Hb (g/L)* | *WBC ($10^9$/L)* | *ANC ($10^9$/L)* | *Platelets ($10^9$/L)* | *Treatment* | *Included in Immunophenotyping experiments* | *Included in computational analysis* | *Included in survival analysis* | *Included in cell cycle analysis* |
|---|---|---|---|---|---|---|---|---|---|---|---|---|---|---|---|---|---|
| MDS137 | 01 | M | 78 | MDS-AML | HIGH | 45,XY,-7[17]/46,XY[7] | 20.5 | 20 | 126 | 2.1 | 0.7 | 64 | No | Yes | Yes | Yes | No |
| MDS324 | 01 | F | 76 | CMML-2 | HIGH | 45,XX,-7[9]/46,XX[21] | 18.0 | 100 | 78 | 38.9 | 22.6 | 71 | No | Yes | Yes | Yes | Yes |
| MDS433 | 01 | M | 39 | RAEB-2 | HIGH | 45,XX,-7[14]/46,XX[7] | 12.0 | 30 | 85 | 2.2 | 0.7 | 29 | No | Yes | No | No | No |
| MDS260 | 03 | M | 69 | RCMD-RS | INT-2 | 47,XY,-7[16]/46,XY[8] | 3 | 50 | 110 | 2.4 | 1.2 | 71 | Azacitidine | Yes | Yes | Yes | Yes |
| | 04 | M | 70 | RAEB-1 | INT-2 | 47,XY,-7[16]/46,XY[8] | 8.5 | 60 | 113 | 2.5 | 1.2 | 21 | Azacitidine | Yes | No | No | No |
| MDS297 | 01 | M | 76 | RAEB-1 | INT-2 | 45,XY,-7[19]/45,X-Y[2]/46,XY[4] | 6.5 | 70 | 96 | 10 | 6.2 | 123 | No | No | Yes | Yes | No |
| MDS342 | 01 | M | 70 | RAEB-1 | INT-2 | 45,XY,-7[24]/46,XY[1] | 5.5 | 50 | 79 | 3.8 | 1.9 | 11 | No | Yes | Yes | Yes | Yes |
| | 02 | M | 70 | RAEB-1 | INT-2 | 46,XY,-7[24]/46,XY[1] | 2.0 | N/A | 76 | 1.5 | 0.5 | 9 | Azacitidine + Eltrombopag | Yes | No | No | No |
| MDS381 | 01 | F | 75 | RCMD | INT-2 | 45,XX,-7[23]/46,XX[2] | 4.5 | 40 | 91 | 1.6 | 1.2 | 29 | No | Yes | Yes | Yes | No |
| | 02 | F | 75 | RCMD | INT-2 | 45,XX,-7[23]/46,XX[2] | 4.0 | 30 | 104 | 2.3 | 1.2 | 59 | Azacitidine | Yes | No | No | No |
| MDS372 | 01 | F | 85 | RCMD | INT-1 | 45,XY,-7[25] | 4.0 | 80 | 132 | 15.9 | 10.8 | 196 | No | Yes | Yes | Yes | Yes |

**MDS non -7**

| Patient ID | Visit | Sex | Age | WHO | IPSS | Cytogenetics | BM Blast (%) | BM Cellula rity (%) | Hb (g/L) | WBC ($10^9$/L) | ANC ($10^9$/L) | Platelets ($10^9$/L) | Treatment | Included in Immunophenotyping experiments |
|---|---|---|---|---|---|---|---|---|---|---|---|---|---|---|
| MDS397 | 01 | M | 75 | RAEB-2 | INT-2 | 45,X-Y[25] | 12.0 | 40 | 113 | 2.0 | 0.2 | 52 | No | Yes |
| MDS292 | 01 | F | 78 | RAEB-2 | INT-2 | 46,XX[28] | 15.0 | 80 | 106 | 1.9 | N/A | 288 | No | Yes |
| MDS470 | 01 | M | 81 | RAEB-2 | INT-2 | 46,XY[20] | 15.5 | 100 | 89 | 10.8 | 4.9 | 296 | No | Yes |
| MDS472 | 01 | M | 84 | MDS-AML | HIGH | 46,XY[25] | 26.5 | 90 | 119 | 116.2 | 55.8 | 38 | No | Yes |
| MDSSW09 | 01 | F | 49 | RAEB-1 | INT-2 | 45,X-X,del(5)(13q33)[17]/46,idem+mar[3]/46,XX[5] | 10.5 | NA | 94 | 5.9 | 2.7 | 40 | No | Yes |
| MDSSW16 | 01 | F | 85 | RAEB-2 | INT-2 | 46,XX,del(5)(q13q33)[3]/44-45,XX,del(5)(q13q33),der(7)t(7;12)(q21?,?),-12,der(18)t(12;18)(?;q)[5]/46,XX[15] | 10.0 | 80 | 98 | 3.4 | 1.7 | 38 | No | Yes |

Abbreviations used: WHO, world health organization; IPSS, international prognostic scoring system; WBC, white blood cell; Hb, hemoglobin; ANC, absolute neutrophil count; BM, Bone Marrow; F, female; M, male; RCMD, refractory cytopenia with multilineage dysplasia; RCMD-RS, refractory cytopenia with multilineage dysplasia with ringed sideroblasts; RAEB, refractory anemia with excess blasts; MPN, myeloproliferative neoplasm; MDS-AML, secondary AML from MDS; CMML, chronic myelomonocytic leukemia; INT, Intermediate. N/A, not applicable.

**Supplemental Table 2. Co-occurrence of TP53 with Complex karyotype.**

| | Complex karyotype with -7/del(7q) | Complex karyotype without -7/del(7q) |
|---|---|---|
| **TP53 occurence** | 5/9 | 6/34 |
| **TP53 frequency** | 55,55% | 17,60% |

The data for the Complex karyotype without -7/del(7q) were extracted from the meta-analysis Sanger Institute cohort [1]. Fisher exact test of the two groups showed a statistically significant difference with a two-tailed P value equals 0.0338.

**Supplemental Table 3. Processed and re-aligned final targeted sequencing reads from Karolinska Institute cohort.**

| Patient | Mutation or chr7 SNP | Chromosome | Position | ALT | REF |
|---|---|---|---|---|---|
| MDS324 | rs2072407 | 7 | 148508833 | 1279 | 8 |
| | rs10274535 | 7 | 148543525 | 295 | 1 |
| | ASXL1_Y591X | 20 | 31022288 | 859 | 750 |
| | IDH2_R140Q | 15 | 90631934 | 1054 | 1110 |
| | JAK2_V617F | 9 | 5073770 | 593 | 33 |
| | SRSF2_P95H | 17 | 74732959 | 630 | 779 |
| MDS372 | rs2072407 | 7 | 148508833 | 51 | 401 |
| | rs28723387 | 7 | 148543753 | 14 | 142 |
| | rs73158267 | 7 | 148544633 | 91 | 12 |
| | rs10274535 | 7 | 148543525 | 6 | 86 |
| | IDH2_R140Q | 15 | 90631934 | 687 | 919 |
| MDS137 | rs2072407 | 7 | 148508833 | 50 | 271 |
| | rs10268879 | 7 | 148506363 | 272 | 27 |
| | rs41277437 | 7 | 148511171 | 292 | 2862 |
| | U2AF1_Q157P | 21 | 44514777 | 1472 | 1956 |
| MDS297 | rs2072407 | 7 | 148508833 | 1735 | 432 |
| | rs10274535 | 7 | 148543525 | 725 | 221 |
| | rs41277437 | 7 | 148511171 | 5841 | 1850 |
| | IDH2_R140Q | 15 | 90631934 | 4078 | 6453 |
| MDS260 | rs2072407 | 7 | 148508833 | 700 | 254 |
| | rs28723387 | 7 | 148543753 | 418 | 134 |
| | rs73158267 | 7 | 148544633 | 23 | 83 |
| | rs10274535 | 7 | 148543525 | 278 | 69 |
| | rs183343901 | 7 | 148512231 | 40 | 111 |
| | SRSF2-P95H | 17 | 74732959 | 722 | 1232 |
| MDS109 | rs2072407 | 7 | 148508833 | 333 | 148 |
| | rs10274535 | 7 | 148543525 | 30 | 124 |
| | rs183943237 | 7 | 148506293 | 54 | 291 |
| | SRSF2-P95H | 17 | 74732959 | 305 | 592 |
| | JAK2_V617F | 9 | 5073770 | 348 | 155 |

Raw reads from the identified mutations and SNPs in chromosome 7 located in the *EZH2* gene locus. SNP, Single nucleotide polymorphism; ALT, total number of reads for alternative allele or variant; REF, total number of reads for reference allele.

**Supplemental Table 4. Prediction of order of genomic lesions in -7/del(7q) patients**

| Patient ID | gene | order | mut.fraction.lower.CI | mut.fraction.median | mut.fraction.upper.CI | chr7.fraction.lower.CI | chr7.fraction.median | chr7.fraction.upper.CI |
|---|---|---|---|---|---|---|---|---|
| PD6223 | NRAS | chr7.greater | 0,496 | 0,576 | 0,656 | 0,890 | 0,899 | 0,908 |
| PD6543 | CBL | chr7.greater | 0,380 | 0,456 | 0,537 | 0,597 | 0,612 | 0,627 |
| PD6890 | ASXL1 | chr7.greater | 0,254 | 0,388 | 0,522 | 0,699 | 0,720 | 0,740 |
| PD6228 | KIT | mut.greater | 0,984 | 1,072 | 1,160 | 0,691 | 0,706 | 0,721 |
| PD6228 | TET2 | chr7.greater | 0,445 | 0,506 | 0,566 | 0,691 | 0,706 | 0,721 |
| MDS297 | IDH2 | mut.greater | 0,756 | 0,774 | 0,793 | 0,685 | 0,698 | 0,712 |
| MDS260 | SRSF2 | mut.greater | 0,803 | 0,842 | 0,881 | 0,641 | 0,675 | 0,708 |
| MDS109 | SRSF2 | incoclusive | 0,655 | 0,715 | 0,774 | 0,641 | 0,690 | 0,734 |
| MDS109 | JAK2 | mut.greater | 0,732 | 0,779 | 0,824 | 0,641 | 0,690 | 0,734 |
| PD6941 | TET2 | chr7.greater | 0,384 | 0,456 | 0,532 | 0,531 | 0,556 | 0,579 |
| PD6941 | U2AF1 | mut.greater | 0,721 | 0,902 | 1,082 | 0,531 | 0,556 | 0,579 |
| MDS324 | SRSF2 | chr7.greater | 0,843 | 0,894 | 0,945 | 0,991 | 0,995 | 0,998 |
| MDS324 | ASXL1 | inconclusive | 1,021 | 1,068 | 1,116 | 0,991 | 0,995 | 0,998 |
| MDS324 | IDH2 | inconclusive | 0,932 | 0,974 | 1,018 | 0,991 | 0,995 | 0,998 |
| MDS324 | JAK2 | chr7.greater | 0,930 | 0,951 | 0,986 | 0,991 | 0,995 | 0,998 |
| PD6335 | PTPN11 | chr7.greater | 0,696 | 0,780 | 0,868 | 0,906 | 0,914 | 0,922 |
| PD6335 | SFRS2 | inconclusive | 0,655 | 0,897 | 1,138 | 0,906 | 0,914 | 0,922 |
| MDS137 | U2AF1 | inconclusive | 0,851 | 0,883 | 0,916 | 0,880 | 0,892 | 0,903 |
| MDS372 | IDH2 | inconclusive | 0,805 | 0,853 | 0,900 | 0,858 | 0,885 | 0,910 |
| PD6515 | U2AF1 | inconclusive | 0,545 | 0,681 | 0,817 | 0,700 | 0,717 | 0,733 |
| PD6958 | IDH1 | inconclusive | 0,540 | 0,624 | 0,708 | 0,676 | 0,693 | 0,709 |
| PD7037 | EZH2 | chr7.greater | 0,117 | 0,151 | 0,187 | 0,783 | 0,800 | 0,815 |
| PD7037 | EZH2 | chr7.greater | 0,389 | 0,455 | 0,523 | 0,783 | 0,800 | 0,815 |
| PD7037 | RUNX1 | incoclusive | 0,676 | 0,845 | 1,014 | 0,783 | 0,800 | 0,815 |
| PD6251 | EZH2 | chr7.greater | 0,623 | 0,714 | 0,801 | 0,867 | 0,878 | 0,889 |
| PD6251 | U2AF1 | incoclusive | 0,791 | 0,919 | 1,047 | 0,867 | 0,878 | 0,889 |

The table summarizes the predicted order of chromosome 7 abnormalities (chr7) and recurrent DNA mutations (mut), based on the fraction of cells determined by sequencing to have the specified genomic lesions, and the specific confidence intervals in each -7/del(7q) case. fraction, fraction of cells; CI, 95% confidence interval.

# SUPPLEMENTAL FIGURES

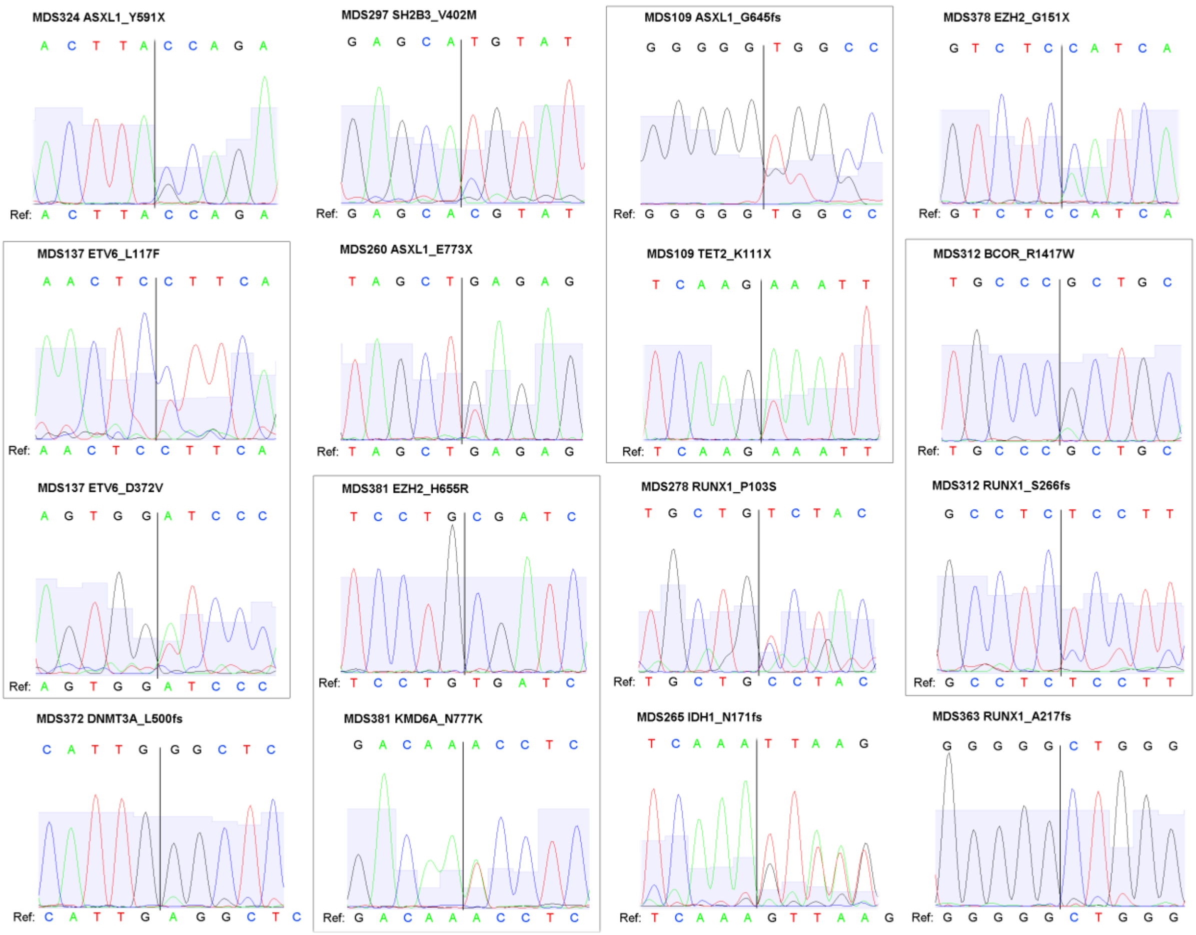


**Supplemental Figure 1. Mutation validation by Sanger sequencing**

All non-recurrent mutations (truncating and unknown) detected by Haloplex were independently validated by Sanger sequencing. The chromatogram shows 5 bases before the nucleotide change and 4 bases after the nucleotide changes. The reference sequences are indicated on the bottom and the raw sequences of patients' mononuclear cells are shown on the top. The targeted base change is indicated by the vertical line.

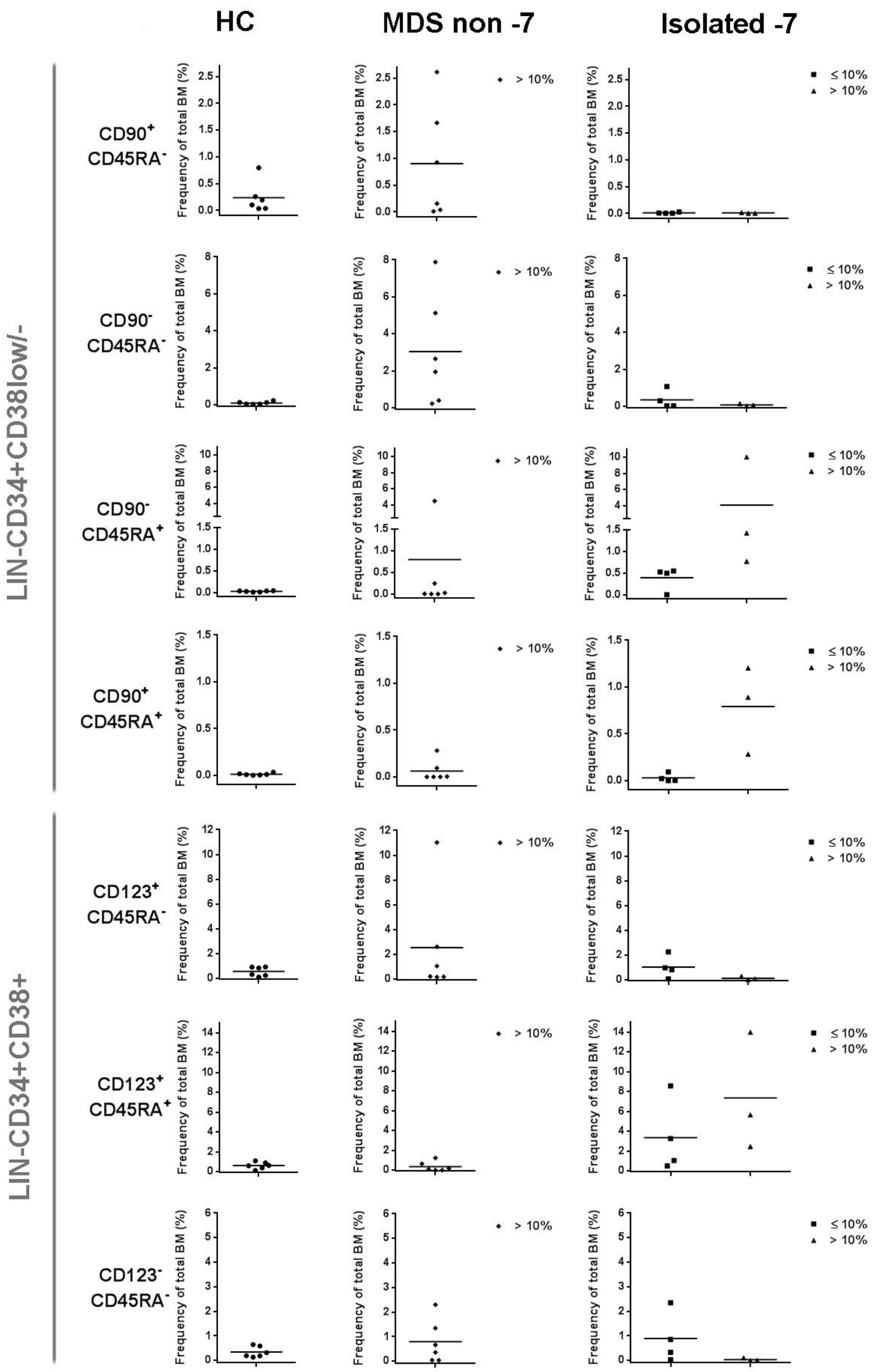


**Supplemental Figure 2. Frequency of stem and progenitor cells within total BM**

Healthy controls (HC), n=6; int-2/high risk IPSS MDS patients without -7 (MDS non -7), n=6; Isolated -7, n=7. Isolated -7 patients were sub-grouped further based on the percentage of BM blasts (≤10% and >10%). Each dot represents an individual patient and the line represents the mean in each group.

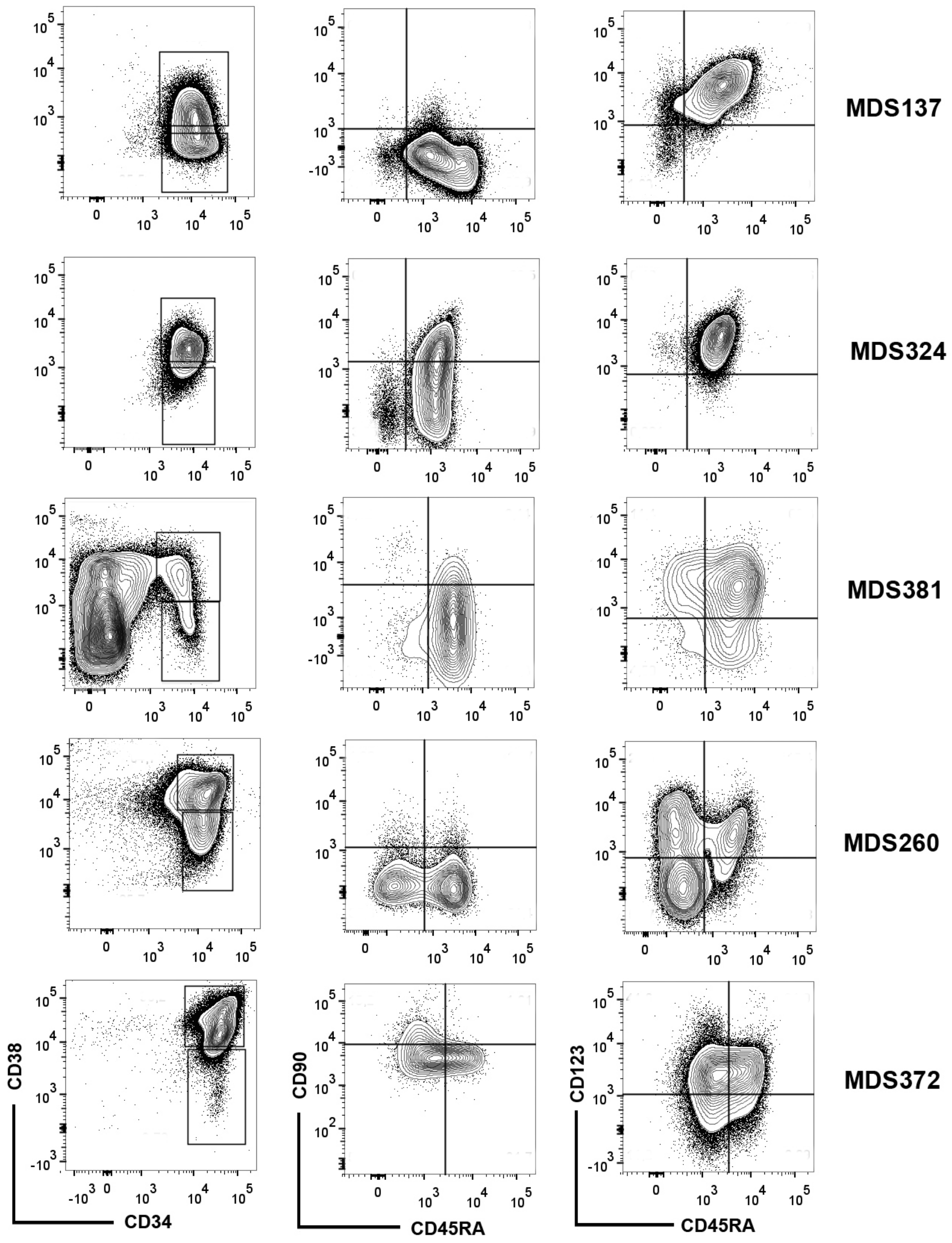


**Supplemental Figure 3. Stem and progenitor cell profiles for isolated -7 MDS patients.** FACS analysis of phenotypic hematopoietic stem and progenitor compartments in remaining five isolated -7 MDS patients. Plots in the first column were previously gated on LIN$^-$, in the second column were previously gated on LIN$^-$CD34$^+$CD38$^{low/-}$ and in the third column were previously gated on LIN$^-$CD34$^+$CD38$^+$.

| | | MDS381 | MDS342 | MDS137 | | MDS297 | | MDS260 | | MDS372 | | MDS324 | | | |
|---|---|---|---|---|---|---|---|---|---|---|---|---|---|---|---|
| | | -7 | -7 | -7 | U2AF1-Q157R | -7 | IDH2-R140Q | -7 | SRSF2-P95H | -7 | IDH2-R140Q | -7 | SRSF2-P95H | IDH2-R140Q | JAK2-V617F |
| $LIN^{-}$ $CD34^{+}$ $CD38^{low/-}$ | $CD90^{+}CD45RA^{-}$ | + | N/A | N/A | N/A | N/A | N/A | N/A | N/A | Low | + | N/A | N/A | N/A | N/A |
| | $CD90^{-}CD45RA^{-}$ | + | + | + | + | + | + | + | + | + | + | + | + | + | + |
| | $CD90^{-}CD45RA^{+}$ | + | + | + | + | + | + | + | + | + | + | + | + | + | Low |
| | $CD90^{+}CD45RA^{+}$ | + | + | + | + | N/A | N/A | N/A | N/A | N/A | N/A | + | + | Low | Low |
| $LIN^{-}$ $CD34^{+}$ $CD38^{+}$ | $CD123^{+}CD45RA^{-}$ | + | + | + | + | + | + | + | + | + | + | + | + | + | + |
| | $CD123^{+}CD45RA^{+}$ | + | + | + | + | + | + | + | + | + | + | + | + | + | Low |
| | $CD123^{-}CD45RA^{-}$ | + | + | + | + | + | + | + | + | + | + | + | + | + | Low |

**Supplemental Figure 4. Detection of recurrent oncogenic genetic lesions in purified stem and progenitor subsets.**

Targeted sequencing and FISH analysis for selected oncogenic lesions in purified stem and progenitor cell populations. Dark blue color (+) indicates confident detection of genetic lesion investigated ( > 10% cells containing -7 by FISH or DNA oncogenic mutations by Fluidigm SNP assay). Light Blue (Low) indicates low or non-confident detection of genetic lesions ( < 10% of cells as described in Supplemental methods; or less < 10% cells containing -7 by FISH). N/A: not applicable; cell populations not available for analysis.

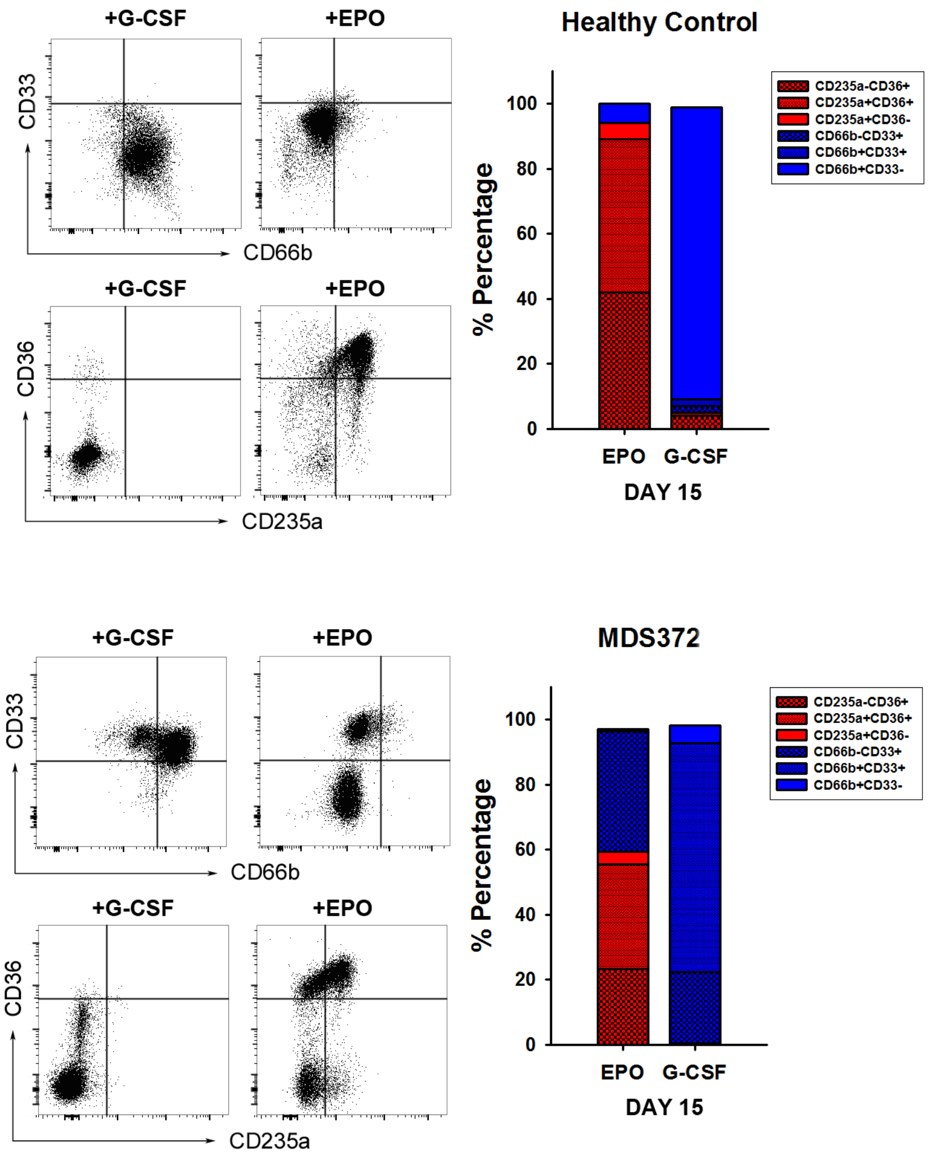


**Supplemental Figure 5. Myeloid and erythroid differentiation culture.**

Representative FACS profiles after 15 days of culture of CD34$^{+}$ cells from healthy control (top) and monosomy 7 MDS patient MDS372 (bottom) in conditions supporting myeloid (G-CSF) and erythroid differentiation (EPO). Enumeration of frequency, as percentage of total live cells, for erythroid compartments (CD235a$^{-}$CD36$^{+}$, CD235a$^{+}$CD36$^{+}$, CD235a$^{+}$CD36$^{-}$), and myeloid compartments (CD66b$^{-}$CD33$^{+}$, CD66b$^{+}$CD33$^{+}$, CD66b$^{+}$CD33$^{-}$).

## SUPPLEMENTAL REFERENCES

1. Papaemmanuil E, Gerstung M, Malcovati L, Tauro S, Gundem G, Van Loo P, Yoon CJ, Ellis P, Wedge DC, Pellagatti A, Shlien A, Groves MJ, Forbes SA, et al. Clinical and biological implications of driver mutations in myelodysplastic syndromes. Blood. 2013; 122(22):3616-3627.

2. Lee WP, Stromberg MP, Ward A, Stewart C, Garrison EP and Marth GT. MOSAIK: a hash-based algorithm for accurate next-generation sequencing short-read mapping. PLoS One. 2014; 9(3):e90581.

3. McKenna A, Hanna M, Banks E, Sivachenko A, Cibulskis K, Kernytsky A, Garimella K, Altshuler D, Gabriel S, Daly M and DePristo MA. The Genome Analysis Toolkit: a MapReduce framework for analyzing next-generation DNA sequencing data. Genome research. 2010; 20(9):1297-1303.

4. Kumar P, Henikoff S and Ng PC. Predicting the effects of coding non-synonymous variants on protein function using the SIFT algorithm. Nat Protoc. 2009; 4(7):1073-1081.

5. Stephens PJ, Tarpey PS, Davies H, Van Loo P, Greenman C, Wedge DC, Nik-Zainal S, Martin S, Varela I, Bignell GR, Yates LR, Papaemmanuil E, Beare D, et al. The landscape of cancer genes and mutational processes in breast cancer. Nature. 2012; 486(7403):400-404.

6. Bolli N, Avet-Loiseau H, Wedge DC, Van Loo P, Alexandrov LB, Martincorena I, Dawson KJ, Iorio F, Nik-Zainal S, Bignell GR, Hinton JW, Li Y, Tubio JM, et al.

Heterogeneity of genomic evolution and mutational profiles in multiple myeloma. Nature communications. 2014; 5:2997.

7. Tehranchi R, Woll PS, Anderson K, Buza-Vidas N, Mizukami T, Mead AJ, Astrand-Grundstrom I, Strombeck B, Horvat A, Ferry H, Dhanda RS, Hast R, Ryden T, et al. Persistent malignant stem cells in del(5q) myelodysplasia in remission. N Engl J Med. 2010; 363(11):1025-1037.

8. Maecker HT and Trotter J. Flow cytometry controls, instrument setup, and the determination of positivity. Cytometry A. 2006; 69(9):1037-1042.

9. Hogge DE, Lansdorp PM, Reid D, Gerhard B and Eaves CJ. Enhanced detection, maintenance, and differentiation of primitive human hematopoietic cells in cultures containing murine fibroblasts engineered to produce human steel factor, interleukin-3, and granulocyte colony-stimulating factor. Blood. 1996; 88(10):3765-3773.

10. Nikpour M, Pellagatti A, Liu A, Karimi M, Malcovati L, Gogvadze V, Forsblom AM, Wainscoat JS, Cazzola M, Zhivotovsky B, Grandien A, Boultwood J and Hellstrom-Lindberg E. Gene expression profiling of erythroblasts from refractory anaemia with ring sideroblasts (RARS) and effects of G-CSF. Br J Haematol. 2010; 149(6):844-854.

11. Conte S, Katayama S, Vesterlund L, Karimi M, Dimitriou M, Jansson M, Mortera-Blanco T, Unneberg P, Papaemmanuil E, Sander B, Skoog T, Campbell P, Walfridsson J, et al. Aberrant splicing of genes involved in haemoglobin synthesis and impaired terminal erythroid maturation in SF3B1 mutated refractory anaemia with ring sideroblasts. Br J Haematol. 2015; 171(4):478-490.

12. Woll PS, Kjallquist U, Chowdhury O, Doolittle H, Wedge DC, Thongjuea S, Erlandsson R, Ngara M, Anderson K, Deng Q, Mead AJ, Stenson L, Giustacchini A, et al. Myelodysplastic syndromes are propagated by rare and distinct human cancer stem cells in vivo. Cancer Cell. 2014; 25(6):794-808.
